# Food Sources of Energy and Nutrients of Public Health Concern and Nutrients to Limit with a Focus on Milk and other Dairy Foods in Children 2 to 18 Years of Age: National Health and Nutrition Examination Survey, 2011–2014

**DOI:** 10.3390/nu10081050

**Published:** 2018-08-09

**Authors:** Carol E. O’Neil, Theresa A. Nicklas, Victor L. Fulgoni

**Affiliations:** 1 Louisiana State University Agricultural Center (Emeritus), School of Nutrition and Food Science, 143 Kenilworth Parkway, Baton Rouge, LA 70808, USA; VIC3rd@aol.com; 2 USDA/ARS/CNRC, 1100 Bates St., Baylor College of Medicine, Houston, TX 77030, USA; tnicklas@bcm.edu; 3 Nutrition Impact, LLC, 9725 D Drive North, Battle Creek, MI 49014, USA

**Keywords:** NHANES, energy intakes, nutrients, children, adolescents, dietary sources, dairy foods

## Abstract

Many children are not meeting current nutrient recommendations. The objective of this study was to determine the food sources of energy, nutrients of public health concern, and nutrients to limit with a focus on dairy foods. Twenty-four-hour dietary recall data from children 2–5 (*n* = 1511), 6–11 (*n* = 2193), and 12–18 years (*n* = 2172) participating in NHANES 2011–2014 were analyzed. Energy, fiber, calcium, potassium, vitamin D, added sugars, saturated fatty acids (SFA), and sodium intakes were sample-weighted and ranked on percentage contribution to the diet using specific food group intake and disaggregated data for dairy foods. For children 2–5, 6–11, and 12–18 years, milk, sweet bakery products, and sweetened beverages, respectively were the top food sources of energy, respectively. For calcium, potassium, and vitamin D, milk was the top ranked food source in all age groups. For children 2–5, 6–11, and 12–18 years, milk, sweet bakery products, and pizza, respectively were the top three ranked food sources of SFA; and sugar sweetened beverages and sweet bakery products were to top two food group sources of added sugars. Cured meats/poultry, pizza, and pizza, respectively, were the top ranked food sources of sodium for the three age groups. Identification of food sources of these nutrients can help health professionals implement appropriate dietary recommendations and plan age-appropriate interventions.

## 1. Introduction

Dairy products are rich in three of the four nutrients of public health concern: calcium, vitamin D, and potassium [1]. The 2015–2020 Dietary Guidelines Advisory Committee (DGAC) determined that several nutrients: vitamins A, E, and C; folate; magnesium; and iron (in adolescent females) were under consumed relative to the Estimated Average Requirement (EAR) or Adequate Intake (AI) levels set by the Institute of Medicine and these were characterized as “shortfall nutrients”. The DGAC confirmed that fiber, calcium, vitamin D, and potassium remained nutrients of public health concern since underconsumption has been linked to adverse health outcomes [2]. In children 2–18 years of age (years), milk has previously been shown to be the primary source of calcium, vitamin D, and potassium [3]. Dairy products, especially milk and yogurt, also provide protein, saturated fatty acids (SFA), riboflavin, vitamin B_12_, and phosphorus. 

Modeling techniques with data from the National Health and Nutrition Examination Survey (NHANES) have shown that the prevalence of inadequate calcium and potassium intakes could be reduced if additional servings of dairy foods were consumed [4,5]. However, per captia consumption of fluid milk has declined sharply since 1975, when it was 247 pounds per person, to 154 pounds per person in 2016. The lack of nutrients from fluid milk has been partially offset by an increase in cheese and yogurt consumption over this time span [6]. 

Although dairy foods contribute to shortfall nutrients, there is concern that these foods may also contribute high levels of energy, added sugars, from flavored milk and sweet dairy drinks; SFA; and sodium—all of which may lead to chronic diseases in older adolescents and adults [7,8]. There has been some speculation that plant-based drinks may provide health benefits over dairy foods. However, modeling studies, using NHANES 2007–2010 data, have shown that when comparing the usual intake of macronutrients and shortfall nutrients of three dietary scenarios that increased intake of: (1) plant-based foods; (2) protein-rich plant foods; and (3) dairy foods, including milk, cheese, and yogurt. The dairy model reduced the percent of children not meeting the EAR for calcium, vitamins A and D, magnesium, and protein, while sodium and SFA intakes increased [9]. Thus, it is very important to understand more fully the food sources that provide these important sources of nutrients in dairy; this can be done in part by disaggregating the data.

To help assuage nutrient shortfalls, the recommendation for daily dairy intake for children is age dependent: 2 cup equivalents (CE) for children 2–3 years of age (years), 2.5 CE for children 4–8 years, and 3 CE for children 9–18 years [10]. In general, young children meet the recommendations for dairy; on average, males and females 2–5 years consume 2.04 and 2.03 CE of total dairy, respectively [11]. However, as children get older, consumption goes down, especially in females. Males and females 6–11 years consume 2.53 and 1.90 CE, respectively; and males and females 12–19 years consume 2.40 and 1.61 CE, respectively [11]. These data are concerning, especially for females, since although the recommendation for dairy intake does not change with gender, in general females need [12] and consume [13] less energy than males. In addition, it is concerning that consumption declines with age. 

Understanding food sources of energy, shortfall nutrients, and nutrients to limit is important at any age, including children. Dietary influences and eating behaviors established in childhood play an important role in growth and development in children [14,15]. They also provide a reasonable basis for adult dietary preferences [16]. Although the majority of information linking diet to chronic disease is available for adults, there is some evidence suggesting that encouraging consumption of foods that provide shortfall nutrients while reducing nutrients to limit may reduce risk factors for chronic diseases, including cardiovascular disease (CVD) [17], hypertension [18], insulin sensitivity [19], obesity [20], and abdominal adiposity [21].

Identifying food sources, including mixed-dish foods—such as pizza or Mexican dishes—that provide energy, shortfall nutrients, and nutrients to limit can help nutrition educators design age-specific programs to help them modify food and nutrient intake [22,23]. Targeted nutrition education may have an indirect positive effect of increasing children’s intakes of food groups that provide shortfall nutrients, while limiting foods that provide nutrients to limit, thus moving children closer to meeting dietary recommendations [10,24]. The purpose of this study was to examine food sources providing energy, shortfall nutrients, and nutrients to limit in three age groups of children using data from the NHANES 2011–2014. This is the first detailed list of food sources in children (2–18 years) since the NHANES 2003–2006 studies [3] and is the first to examine food sources in three age groups of children. Additionally, given milk, cheese, and yogurt are used as ingredients in many mixed dishes, which are not captured in a simple analyses of food sources of nutrients, this study also determined the nutrients from milk, cheese, and yogurt in mixed dishes thereby obtaining a more thorough contribution of dairy products to the diet of children.

## 2. Materials and Methods 

### 2.1. Study Overview, Study Population, and Analytic Sample

The NHANES is a program of studies designed to assess the health and nutritional status of free-living individuals in the US. Online information about the NHANES, including the purpose [25], plan and operations, sampling and weighting procedures, analytic guidelines [26], response rates, and population totals [27], is available. Data from children 2–18 years of age (years) participating in the NHANES from 2011 to 2014 were used for these analyses. The final analytic sample had 5876 participants; children were separated into three age groups: 2–5 years (*n* = 1511), 6–11 years (*n* = 2193), and 12–18 years (*n* = 2172). The National Center for Health Statistics (NCHS) Research Ethics Review Board has approved the use of human subjects for NHANES studies [28]; and further institutional review was not required.

### 2.2. Dietary Intake 

Dietary intake data for the NHANES used in this study were obtained from the in-person 24-h dietary recall interview [29] using an Automated Multiple-Pass Method [30]. Although a second, telephone interview, was also taken 3 to 10 days after the in-person interview, only the in-person interview was used because of the difference in the methodology for collecting the two recalls. A single 24-h dietary recall administered in a large population can provide data to adequately estimate population mean intakes [31]. Survey participants 12 years and older completed their own dietary interview; children 6 to 11 years were assisted by an adult, usually a parent; and parents/guardians reported for children younger than 6 years [29]. 

### 2.3. Food Groupings and Composition

The relevant What We Eat in America (WWEIA), the dietary component of NHANES, food category classification systems [32] were used to classify all foods. The WWEIA food categories contain 15 main groups: milk and dairy; protein foods; mixed dishes; grains; snacks and sweets; fruit; vegetables; beverages, nonalcoholic; alcoholic beverages; water; fats and oils; condiments and sauces; sugars; infant formula and baby food; and other. The WWEIA food categories also consists of 47 subgroups. For example, for the milk and dairy main group, the subgroups were milk, flavored milk, cheese, dairy drinks and substitutes, and yogurt. For these analyses we focused on the 47 subgroups.

Using the relevant Food Patterns Equivalent Database [33] milk, cheese, and yogurt servings of non-dairy foods and especially mixed dishes were determined. The nutrient composition in the relevant Food and Nutrient Database for Dietary Studies FNDDS 2011–2012 and 2013–2014 [34] linked to SR 26 and SR 28 respectively [1] for milk, NFS (not further specified); cheese, NFS; and yogurt, NFS was used to assess energy and nutrient contribution of dairy servings non-dairy foods. The nutrients reported herein are the nutrients of public health concern [2]: dietary fiber, calcium, vitamin D, and potassium; and nutrients to limit: SFA, added sugars, and sodium. 

Data are reported as specific food group (SFG) intake, adjusted intake, and delta intake. Specific food group intake is intake from the dairy food groups (milk, cheese, and yogurt). Adjusted intake is the total daily intake after nutrients from dairy from non-dairy foods (e.g., mixed dishes) have been included, and reflect the disaggregation. Delta intake is the amount of nutrients from dairy in non-dairy foods that was added to or removed from the specific food group intake to calculate the adjusted intake. The consumer number (*n*) for delta was the number of subjects that consumed dairy from mixed dishes. 

### 2.4. Statistical Analyses

Data were analyzed using SAS 9.2 and SUDAAN release 11.0 (Research Triangle Institute, Research Triangle Park, NC, USA) with survey parameters including strata, primary sampling units, and dietary sample weights [26]. Means and standard errors (SE) of energy and nutrient intakes from the total diet and from each food group were determined using PROC DESCRIPT of SUDAAN. Percentages of total energy and nutrient intakes from each food group were calculated from the average consumption of each food. Mean intakes were tabulated by ranked order to 1% of consumption. 

## 3. Results

### 3.1. Contribution of Foods to Percent Energy Intake

Total mean daily energy consumption was 1535 ± 19 kcals ± SE; 1953 ± 23.0 kcals; and 2056.0 ± 33.2 kcals, respectively for children 2–5, 6–11, and 12–18 years, respectively. Table 1 shows the food sources contributing at least 1% of percent energy intake from the WWEIA sub-categories. There were 31, 29, and 31 food sources that contributed at least 1% of SFG energy intake of children 2–5, 6–11, and 12–18 years, respectively. Using SFG intake data (kcals; % of energy) for children 2–5 years, milk (136 kcals; 8.9% of energy), sweet bakery products (116 kcals; 7.6%), and grain-based mixed dishes (86 kcals; 5.6%) were ranked as the top food sources of energy. Cheese was ranked as the 18th food source of energy (39 kcals; 2.5%). Using adjusted (disaggregated) data, milk and sweet bakery products remained the two top ranked foods (151 kcals; 9.8% and 115 kcals; 7.5%) with mean delta intakes of +15 and −1 kcals, respectively. Cheese was the fifth ranked energy source (74 kcals; 4.8%).

The top SFG sources of energy for children 6–11 years were sweet bakery products (164 kcals; 8.4%), pizza (132 kcals; 6.8%), and sweetened beverages (112 kcals; 5.7%), with milk and cheese ranked 7th (96 kcals; 4.9%) and 21st (38 kcals; 1.9%), respectively. Using adjusted data, sweet bakery products and milk ranked first (163 kcals; 8.3%) and second (117 kcals; 6.0%), respectively, with mean delta intakes of −2 and +21 kcals.

For children 12–18 years, the top SFG intakes of food sources were sweetened beverages (162 kcals; 7.9%), sweet bakery products (139 kcals; 6.8%), and pizza (135 kcals; 6.6%), with milk and cheese ranked 7th (96 kcals; 4.7%) and 18th (42 kcals; 2.1%), respectively. Using adjusted data sweetened beverages and sweet bakery products continued to rank first (160 kcals; 7.8%) and second (137 kcals; 6.7%); respectively; each with a delta value of −1 kcals. Milk (117 kcals; 5.7%) and cheese (118 kcals; 5.7%) were the fourth and fifth ranked food groups, with mean delta intakes of +21.5 kcals and +74 kcals.

### 3.2. Contribution of Foods to Percent Fiber Intake

Total mean daily dietary fiber intake was 11.8 ± 0.2 g; 14.6 ± 0.3 g; and 14.7 ± 0.3 g, for children 2–5, 6–11, and 12–18 years, respectively. Table 2 shows the food sources contributing to at least 1% of daily fiber intake. In all three age groups there were 22 different food groups that contributed at least 1% of fiber intake. For the specific food group intakes, fruit was the top contributor to fiber intake with a mean of 2 g; 17.2%; 1.9 g; 12.8%, and 1.5 g; 10%, for each age group, respectively. For children 2–5 years, SFG intake was followed by bread, rolls, tortillas (1.2 g; 10%) and ready-to-eat cereal (RTEC) (0.9 g; 7.4%). For children 6–11 years, bread, rolls, tortillas (1.5 g; 10%) and mixed dishes—pizza (1.1%; 7.4%) followed; finally, in children 12–18 years bread, rolls, tortillas (1.5 g; 10%), was followed by mixed dishes—Mexican (1.1 g; 7.7%). There were no differences in rank order after adjustment of any of the foods in all three age groups and delta intake was zero.

### 3.3. Contribution of Foods to Percent Calcium Intake 

Total mean daily calcium intake was 971.5 ± 23.8 mg; 1074.5 ± 19.1 mg; and 1056.9 ± 21.2 for children 2–5, 6–11, and 12–18 years, respectively. Table 3 shows the food sources contributing at least 1% of total calcium intake. There were 20, 19, and 21 food sources that contributed at least 1% of SFG calcium intake of children 2–5, 6–11, and 12–18 years, respectively. Using SFG intake data for children 2–5 years, milk (318 mg; 32.7% of calcium), cheese (99 mg; 10.2%), and flavored milk (74 mg; 7.6%) were ranked as the top food sources of calcium. Using adjusted data, the rank order in children 2–5 years remained the same; however the percentages changed for: milk (352 mg; 36.2%; +34 mg) and cheese (190 mg; 19.5%; +90.6 mg), but not for flavored milk (74 mg; 7.6%; 0.0 mg). 

The top three SFG intake sources of calcium for children 6–11 years were milk (238 mg; 22.2%), cheese (107 mg; 10.0%), and pizza (89 mg; 5.7%). Using adjusted data, milk remained the top source of calcium (287; 26.7%; +49.1 mg), with cheese ranked second (282 mg; 26.2%; +174.6 mg); flavored milk ranked third (88 mg; 8.2%; 0.0 mg). Pizza dropped to the 20th source of calcium (10 mg; 0.9%; +175 mg).

Milk (240 mg; 22.7%), cheese (114 mg; 10.8%), and pizza (86 mg; 8.1%) were the SFG intake top food sources of calcium in children 12–18 years. Using adjusted data, cheese was the top ranked food (301 mg; 28.5%; +187 mg), followed by milk (290 mg; 27.4%; +50 mg); pizza, the third rank food in the SFG data, fell to 23rd (7 mg; 0.7%; −79 mg) and bread, rolls, and tortillas was the third most common source of calcium (56 mg; 5.3%; −1 mg).

### 3.4. Contribution of Foods to Percent Vitamin D Intake

Total mean daily vitamin D intake was 6.2 ± 0.2 mcg; 5.7 ± 0.1 mcg; and 5.3 ± 0.2 mcg, respectively, for children 2–5, 6–11, and 12–18 years. Table 4 shows the food sources contributing at least 1% of vitamin D intake. There were 11, 14, and 14 different SFG sources that contributed at least 1% of the vitamin D intake of children 2–5, 6–11, and 12–18 years, respectively. Using SFG intake data for children 2–5 years, milk (3.3 mcg; 52.6%; 0.2 mcg), flavored milk (0.8 mcg; 12.0%; 0.0 mcg), and (0.5 mcg; 7.5%) were the top food sources of vitamin D. Using adjusted data, the rank order remained the same with milk (3.5 mcg; 55.7%; +0.2 mcg), flavored milk (12 mcg; 12%), and RTEC (7.5 mcg; 7.5%; 0.0 mcg). Eggs were the highest-ranking (fourth) non-fortified SFG food group consumed by this age group (0.3 mcg; 4.4%); however, after adjustment, eggs fell to fifth with a mean of 0.2 mcg; 3.8%.

For children 6–11 years, milk (2.7 mcg; 46.7%), flavored milk (0.9 mcg; 15.2%), and RTEC (0.6 mcg; 9.9%) were the top SFG sources of vitamin D, respectively. Using adjusted data, the rank order remained (2.7 mcg; 46.7%; +0.3 mcg), flavored milk (0.9 mcg; 15.2%; 0.0 mcg), and RTEC (0.6 mcg; 9.9%; 0.0 mcg). Eggs were the highest-ranking non-fortified SFG and adjusted food source of vitamin D (0.2 mcg; 4.1% and 0.2 mcg; 3.5%), respectively.

For children 12–18 years, milk (2.4 mcg; 45.6%), RTEC (0.5 mcg; 9.2%), and flavored milk (0.4 mcg; 7.6%), were the top three SFG food sources of vitamin D. Using adjusted data, milk (2.7 mcg; 51.3%; +0.3 mcg), cheese (0.5 mcg; 9.6%; +0.2 mcg), and RTEC (0.9 mcg; 9.1%; 0.0 mcg) were the top three food sources of vitamin D.

### 3.5. Contribution of Foods to Percent Potassium Intake

Total mean daily potassium intake was 1981.8 ± 39.5 mg; 2197.9 ± 27.0 mg; and 2308.2 ± 44.9 mg for children 2–5, 6–11, and 12–18 years, respectively. Table 5 shows the food sources contributing at least 1% of potassium intake. There were 25, 25, and 26 food sources that contributed at least 1% of potassium intake of children 2–5, 6–11, and 12–18 years, respectively. Using SFG intake data for children 2–5 years, milk (375 mg; 18.9%), fruit (190 mg; 9.6%), and 100% fruit juice (169 mg; 8.5%) were the top food sources of potassium. When the data were adjusted, the rank order of the top food sources of potassium remained the same: milk (417 mg; 21.1%; +41.8 mg), fruit (190 mg; 9.6%; delta 0 mg), and 100% fruit juice (169 mg; 8.5%; delta 0 mg). 

In children 6–11 years, milk (283 mg; 12.9%), fruit (160 mg; 7.3%), and flavored milk (125 mg; 5.7%) were the three SFG top sources of potassium. Using adjusted data, the rank order remained the same with milk (344 mg; 15.6%; +60 mg), fruit (160 mg; 7.3%; −0.1), and flavored milk (125 mg; 5.7%; 0 g) as the top three sources of potassium intake. 

In the oldest group of children, the top SFG food sources of potassium were milk (milk 286 mg; 12.4%), white potatoes (145 mg; 6.3%), and fruit (129 mg; 5.6%). The rank order remained the same for adjusted data, with milk (347 mg; 15%; +61 mg), white potatoes (141 mg; 6.1%; −4 mg), and fruit (129 mg; 5.6%; 0.0 mcg), respectively.

### 3.6. Contribution of Foods to Percent Added Sugars Intake

Total mean daily added sugars intake was 12.0 ± 0.3 teaspoon equivalents (tsp eq) (15.6% of total energy); 18.2 ± 0.4 tsp eq (18.6% energy); and 20.2 ± 0.5 tsp eq (19.6% energy) for children 2–5, 6–11, and 12–18 years, respectively. Table 6 shows the food sources contributing at least 1% of added sugars intake. For SFG intake, 15, 14, and 11 food groups contributed to at least 1% of added sugars intake for the three age groups, respectively. For the youngest group of children, the top three SFG and adjusted food intakes were sweetened beverages (3 tsp eq; 25.3%; 0.0 tsp eq), sweet bakery products (1.9 tsp eq; 16%; 0.0 tsp eq), and other desserts (0.9 tsp eq; 7.5%; 0.0 tsp eq). For children 6–11 years, the top three SFG and adjusted food intakes were sweetened beverages (5.8 tsp eq; 32.1%; 0.0 tsp eq (delta values)), sweet bakery products (2.8 tsp eq; 15.3%; 0.0 tsp eq), and candy (1.5 tsp eq; 8.2%; 0.0 tsp eq). For children 12–18 years, the top three food groups in the SFG and adjusted intakes were sweetened beverages (8.6 tsp eq; 42.5%; 0.0 tsp eq), sweet bakery products (2.4 tsp eq; 11.8%; 0.0 tsp eq), and coffee and tea (1.7 tsp eq; 1.7%; 0.0 tsp eq).

### 3.7. Contribution of Foods to Percent Saturated Fatty Acids (SFA) Intake

Total mean daily SFA intake was 20.1 ± 0.6 g (11.8% total energy); 26.0 ± 0.5 g (12% energy); and 26.0 ± 0.5 g (11.4% energy) for 2–5, 6–11, and 12–18 years, respectively. Table 7 shows the food sources contributing at least 1% of SFA intake. There were 23, 21, and 24 food sources that contributed at least 1% of SFA consumed by children 2–5, 6–11, and 12–18 years, respectively. Using SFG data for children 2–5 years, milk (3.4 g; 16.7%), sweet bakery products (1.8 g; 8.8%) and cheese (1.7 g; 8.2%) were the top sources of dietary SFA. Using adjusted data, the top ranked contributors to SFA intake in children 2–5 years were milk (3.7 g; 18.4%; +0.3 g(delta value)), cheese (3.3 g; 16.4%; 0.0 g), and sweet bakery products (1.8 g; 8.7%; +1.6 g). 

For children 6–11 years, sweet bakery products (2.6 g; 10%), pizza (2.3 g; 9%), and milk (2.2 g; 8.3%) were the top three sources of SFA. Using adjusted data, cheese was the single highest contributor of SFA to the diet (4.7 g; 8.3%; +3.2 g), followed by milk (2.6 g; 10.1%; +0.5 g), and sweet bakery products (2.6 g; 9.9%; 0.0 g). Using these adjusted data, pizza dropped to 11th (0.8 g; 3.2%).

For children 12–18 years, pizza (2.4 g; 9.1%), sweet bakery products (2.2 g; 8.6%), and milk (2.1 g; 8.1%) were the top food sources contributing to SFA intake. Using adjusted data, cheese ranked first (5.1 g; 19.7%; +3.4 g), followed by milk (2.6 g; 10%; +0.5 g), and sweet bakery products (2.2 g; 8.5%; 0.0 g).

### 3.8. Contribution of Foods to Percent Sodium Intake

Total daily mean intake of sodium was 2267.4 ± 37.3 mg; 3036 ± 40.1 mg; and 3394.8 ± 66.6 mg for children 2–5, 6–11, and 12–18 years, respectively. Table 8 shows the food sources contributing at least 1% of sodium intake. There were 28, 28, and 26 food sources that contributed at least 1% of sodium consumed by children 2–5, 6–11, and 12–18 years, respectively. Using SFG data for children 2–5 years, cured meats/poultry (183 mg; 8.1%); grain-based mixed dishes (155 mg; 6.8%); and bread, rolls, and tortillas (144 mg; 6.4%) were the top ranked contributors of sodium to the diet. Using adjusted data, cheese was the top contributor to sodium intake (188 mg; 8.3%; +88 mg); followed by cured meats/poultry (182 mg; 8%; −0.3 mg); and breads, rolls, and tortillas (144 mg; 6.4%; −0.4 mg).

For children 6–11 years, pizza was the top SFG contributor of sodium to the diet (286 mg; 9.4%), followed by Mexican foods (215 mg; 7.1%), and cured meats/poultry (197 mg; 6.5%). Using adjusted data, cheese was the top contributor of sodium to the diet (277 mg; 9.1%; +169 mg), followed by pizza (207 mg; 6.8%; −79 mg), and cured meats/poultry (197 mg; 6.5%).

Using SFG data, pizza was the top contributor of sodium to the diet (297 mg; 8.7%), followed by Mexican foods (224 mg; 6.6%), and cured meats/poultry (223 mg; 6.6%) in children 12–18 years. Using adjusted data, cheese was the top contributor of sodium to the diet (300 mg; 8.8%; +182 mg), followed by cured meats/poultry (221 mg; 6.5%; −0.6 mg), and pizza (216 mg; 6.4%; −80 mg).

## 4. Discussion

This study showed that top food sources contributing to intake of energy, fiber, calcium, vitamin D, potassium, SFA, added sugars, and sodium varied by age group. In addition, food groups providing some of the major sources of nutrients of public health concern also contributed nutrients to limit in the diet. Mixed dishes, especially pizza and Mexican dishes, contributed to the intake of short fall nutrients in the diets of children. 

Nutrients of public health concern [2] have been identified as the shortfall nutrients that pose a substantial risk to the health of our nation. In adults, fiber intake has been associated with a protective effect against gastrointestinal diseases, obesity, CVD, and type 2 diabetes [35]. Fewer studies have been conducted in children; thus, the full impact of dietary fiber intake by children is not clear [36]. Calcium has long been associated with bone and tooth health, but it has also been associated with reduction in the risk of CVD and hypertension; cancers of the colon, rectum, and prostate; kidney stones; and weight management [37]. Potassium is perhaps best recognized for its effect on lowering/controlling blood pressure [38], but other health effects of low potassium intake include a higher risk of stroke [39], insulin resistance, and diabetes [40]. For vitamin D consumption, this study assessed vitamin D3, a prohormone produced in skin through ultraviolet irradiation of 7-dehydrocholesterol, and vitamin D2, found principally in plants [41]. Vitamin D increases intestinal calcium and phosphate absorption, bone calcium mobilization, and the renal reabsorption of calcium, thereby supporting bone mineralization and preventing nutritional rickets in children and osteomalacia in adults [41]. Vitamin D also has other physiologic function, including modulating cell growth, neuromuscular and immune functions, and reducing inflammation [37]. 

For the nutrients of public health concern [2], dairy products, particularly, milk, provided the top source of calcium, vitamin D, and potassium for all age groups. Although dairy foods provided the top sources of most of these nutrients for most age groups in the SFG data, when the data were adjusted, mixed dishes that included dairy products contributed substantially to intake of these nutrients. This suggested that these foods were no longer important sources of calcium, vitamin D, and potassium. For example, in children 6–11 years, the SFG data showed that mixed dishes—pizza was the 3rd top source of calcium; however, after adjustment, mixed dishes—pizza fell to the 20th source as the nutrients from cheese on pizza were reassigned to the cheese food group. In children 12–18 years, mixed dishes—pizza went from the 3rd top source of calcium to the 23rd top source after adjustment. Thus, it is important to recognize that, food groups that contribute nutrients to limit to the diet can also contribute significantly to the intake of nutrients of public health concern.

Although other foods including most dairy foods provide calcium, milk is well established as the principal source of calcium intake by children [3]. The present study, which used disaggregated data, however, clearly showed that dairy foods in mixed dishes and other dishes contributed many of the nutrients of public health concern found in milk. When examining food sources of nutrients in children these other foods should be considered. Milk and other dairy foods are commonly considered to be an important source of dietary potassium and the recommendation changed to 3 CE/day for most age groups in 2005, in part to increase potassium intake [42].

These data clearly demonstrated the importance of fortification of foods with vitamin D. Using adjusted data, fortified foods contributed 75, 72, and 68% of vitamin D intake by the three age groups; with milk/flavored milk contributing the highest amount of dietary vitamin D. These data contrast sharply with a recent study of children in Ireland [43], where milk/yogurt contributed only 13% of dietary vitamin D since most milk in that country is not fortified [43,44]. Since vitamin D increases calcium absorption, the combination of vitamin D and calcium is especially important for bone health. Fortification of milk has been recently reviewed [45]. The 2015–2020 DGAC [2] reconfirmed that vitamin D is a nutrient of public health concern. Data from WWEIA 2013–2014, showed that the intake of vitamin D by children 2–19 years was only 244 IU [13], which is less than half of the 600 IU dietary reference intake recommendation for this age group [37]. Fortification of foods, especially milk and RTEC, is an important way to increase dietary intake of vitamin D. These foods, as well as other foods high in vitamin D, including egg yolks and salmon, should be encouraged. The importance of milk and other dairy foods to potassium intake was clear, as it provided the top source of the nutrient in all three age groups. Potassium intake is very low, with average intakes slightly over half the requirements of most children [13,24]. Thus, high potassium foods including milk and other dairy foods should continue to be encouraged, along with other high potassium foods, notably fruit and vegetables.

Despite the contribution of dairy foods to the intake of shortfall nutrients, including in mixed dishes, there is concern that they contribute high amounts of SFA and sodium to the diet. Using adjusted data, for children 2–5 years, milk, flavored milk, and cheese contributed 39% of SFA (yogurt and dairy drinks/substitutes contributed minimally to SFA in the diet); for children 6–11 milk, flavored milk, and cheese contributed 31% of SFA to the diet; and for children 12–18 years 32% of SFA came from these dairy foods. The DGAC [2] recommends that no more than 10% of energy come from SFA. In this study, the amount of energy from SFA consumed by children varied by age; for children 2–5, 6–11, and 12–18 years the percent energy from SFA was 11.8, 12.0, and 11.4, respectively.

The rationale for the current recommendation is that by reducing SFA, low-density lipoprotein cholesterol levels are reduced and, in turn, the risk for CVD is lowered. Cardiovascular disease, which is the principal cause of death in the world, has its roots in childhood [46]. Recently, however, the relationship between SFA and CVD have been questioned [47,48,49,50], in part due to the nutrients that would replace SFA in the diet [51] and in part because not all food sources of SFA are associated with an unfavorable risk of CVD [52]. A number of studies, including several meta-analyses have shown that consumption of dairy products is associated with a neutral or inverse risk of CVD [53,54,55]. 

One of the easiest ways to reduce the amount of SFA in the diets of children two years and older is to encourage the consumption of low fat milk or flavored milk. When the category description of the milk sub-group was examined, low fat milk contributed only 0.25 g of SFA to the overall intake (data not shown). However, other sources of SFA in the diet also need to be addressed, notably sweet bakery products, such as cookies, brownies, and doughnuts. These foods are also among the principal sources of added sugars to the diet. Reducing the SFA and added sugars content of these foods is more difficult than for milk, since some of the structural integrity and sensory properties of these products are linked to solid fat [56,57]. Thus, consumer education may be the best way to reduce the intake of SFA in the diet.

Cheese was the principal contributor of sodium to the diet in the two older groups of children. Analysis of the contributions of disaggregated food mixtures showed this more clearly than examining the foods “as consumed”. In addition to cheese consumed directly, cheese was an important ingredient in mixed dishes widely consumed by children, including pizza and Mexican dishes. Thus, reducing the sodium in the diet by reducing the amount of cheese consumed may prove difficult. Reformulating cheeses as reduced-sodium products may also be challenging. Not only does salt help prevent microbial growth in cheese [58], low sodium cheeses may not be well received by consumers [59]. A gradual reduction in the amount of salt used in cheese manufacture may help introduce consumers to a lower sodium product [59] or replacement of part of the sodium chloride with potassium chloride [60]. Reduction of cheese in the diet may be an option; however, this would limit the intake of other shortfall nutrients found in cheese.

This study had a number of strengths. The first is was that it used a large, nationally representative sample. The study also demonstrated the differences in food sources of nutrients in three age groups of children. The third is was that disaggregated energy and nutrients from milk, cheese, yogurt, and non-dairy food groups were also considered which gives further insight into the relative contribution of milk, cheese, and yogurt to both nutrients to encourage and to limit. In addition, this approach can help individuals make more informed food choices [61].

The study also had a number of limitations. A limitation is was the use of 24-h dietary recalls to assess intake in NHANES. Participants or proxies relied on memory to self-report dietary intakes; therefore, data were subject to non-sampling errors, including under or over-reporting of energy and foods. The proxies reporting for or assisting children 2–11 years may not know what their children consumed outside of the home [62], which could also result in reporting errors [63]. Concerns about the validity of self-reported dietary intakes in NHANES has led to an ongoing debate about the validity of these data. Some believe strongly that the data are virtually useless [64,65,66] given issues with misreporting, whereas others, including the prestigious National Cancer Center, [2,67,68] use the data recognizing any potential limitations and allow conclusions to be drawn accordingly. According to Ahluwalia [68], the Nutrition Monitoring Advisor for the Division of Health and Nutrition Examination Surveys, NCHS, Centers for Disease Control and Prevention, and coworkers “NHANES collects dietary data in the context of its broad, multipurpose goals”. Their recent review discusses further strengths and limitations of these data. Finally, it should be remembered that cross-sectional studies are used to generate hypotheses, not to test them.

The question may arise as to why “added sugars”, as defined by the Dietary Guidelines for Americans [42] were used in this study rather than the “free sugars” designation used by the World Health Organization [69]. These two terms differ significantly since “free sugars” include those sugars naturally occurring in “…fruit juice”. The authors do not believe that 100% fruit juice, which by definition, as no sugar added should not be considered in the category of free sugars. When evidence-based studies were examined, 100% fruit juice has consistently been shown not to contribute to overweight or obesity in children [70,71,72] or adults [73], instead contributing to nutrient intake and nutrient adequacy, and higher diet quality [71,73,74,75]. Furthermore, since we worked with an American population, it was felt that using the definitions provided by the nutrition policy statement of the US government was more appropriate.

Due to the technical difficulties involved, dairy was disaggregated from mixed dishes only; further insights might be obtained if it had been possible to disaggregate other food groups. Lastly, for this study, the assumption was made that the milk, cheese, and yogurt components of a mixed food follow the nutrient profiles of milk, NFS; cheese, NFS; and yogurt, NFS, but this approach may not provide the best approximation for some foods. For example, some types of cheese in a mix dishes may deviate from having a nutrient content similar to ‘cheese, NFS’ for one or more nutrients.

## 5. Conclusions

This study showed that for children in all three age groups studied, mixed dishes containing dairy foods contributed to calcium, vitamin D, and potassium intake—three of the nutrients of public concern. A caveat of dairy food consumption is that full fat dairy can contribute saturated fatty acids to the diet and cheese, a major component of many of the mixed dishes, such as pizza and Mexican foods, contributes not only saturated fatty acids, but sodium to the diet. The study also showed that fortifying foods with vitamin D was important since few foods contain naturally occurring vitamin D. The study also showed that children, especially those 6–11 and 12–18 years consumed a large proportion of total energy from energy-dense low-nutrient food groups, such as sugar sweetened beverages and sweet bakery products. Those foods contributed little to the nutrients of public health concern, but did contribute to the nutrients to limit, notably added saturated fatty acids and added sugars. Awareness of food and beverage sources of nutrients can help health professionals design and promote effective age-appropriate strategies to increase the nutrient density of the diet. In addition, this awareness can help the food industry to design and market foods frequently consumed by children that are acceptable and lower in energy and nutrients to limit.

## Figures and Tables

**Table 1:** Food/food group sources of mean energy (kcals) intake ^1^ among US children aged 2–18 years (*N* = 5876): National Health and Nutrition Examination Survey 2011–2014.

Mean Energy Intake (kcals) of Children 2–5 Years of Age (*n* = 1511)																	
WWEIA Food Group	Specific Food Group Intake						Adjusted Intake ^2^						Delta Intake				
Sub Group Description	Cons	Rank	Mean	SE	Pct	SE	Cons	Rank	Mean	SE	Pct	SE	Cons	Mean	SE	Pct	SE
Milk	1120	1	136	5.5	8.9	0.3	1415	1	151	5.3	9.8	0.3	1120	15	0.7	1.0	0.1
Sweet Bakery Products	723	2	116	5.3	7.6	0.3	723	2	115	5.5	7.5	0.3	262	−1	0.1	0	0
Mixed Dishes—Grain-based	466	3	86	6.5	5.6	0.4	466	4	79	6.2	5.1	0.4	266	−7	0.7	−0.5	0
Breads, Rolls, Tortillas	818	4	83	6.2	5.4	0.4	818	3	83	6.2	5.4	0.4	30	0	0.1	0	0
100% Juice	755	5	66	4.1	4.3	0.3	755	6	66	4.1	4.3	0.3	0	0	0.0	0	0
Savory Snacks	688	6	65	6.6	4.2	0.4	688	7	65	6.5	4.2	0.4	140	0	0.0	0	0
Fruits	945	7	63	2.3	4.1	0.2	945	8	63	2.3	4.1	0.2	2	0	0.0	0	0
Sweetened Beverages	787	8	61	4.5	4.0	0.3	787	9	61	4.5	4.0	0.3	23	0	0.2	0	0
Poultry	526	9	59	5.1	3.8	0.3	526	10	59	5.1	3.8	0.3	71	0	0.0	0	0
Mixed Dishes—Mexican	201	10	54	6.2	3.5	0.4	201	13	43	4.9	2.8	0.3	189	−11.3	1.4	−0.7	0.1
Mixed Dishes—Pizza	247	11	53	5.9	3.5	0.4	247	17	39	4.2	2.5	0.3	247	−14	1.8	−0.9	0.1
Ready-to-Eat Cereals	695	12	51	2.6	3.3	0.2	695	11	51	2.6	3.3	0.2	12	0	0.0	0	0
Flavored Milk	253	13	48	6.5	3.1	0.4	253	12	48	6.5	3.1	0.4	0	0	0.0	0	0
Quick Breads/Bread Products	293	14	41	5.3	2.7	0.4	293	15	40	5.0	2.6	0.4	274	−2	0.3	−0.1	0
Cured Meats/Poultry	459	15	40	4.0	2.6	0.3	459	14	40	4.0	2.6	0.3	2	0	0.1	0	0
Crackers	353	16	40	3.9	2.6	0.3	353	16	39	3.9	2.6	0.3	86	0	0.1	0	0
Mixed Dishes—Sandwiches	208	17	39	4.0	2.6	0.3	208	18	37	3.9	2.4	0.2	67	−2	0.4	0	0
Cheese	542	18	39	5.3	2.5	0.3	1047	5	74	5.7	4.8	0.4	820	36	1.8	2.3	0.1
Candy	507	19	37	2.7	2.4	0.2	507	19	35	2.6	2.3	0.2	181	−1	0.2	−0.1	0
Other Desserts	416	20	37	3.6	2.4	0.2	416	21	32	3.2	2.1	0.2	291	−5	0.6	−0.3	0
White Potatoes	401	21	35	3.7	2.3	0.2	401	20	34	3.7	2.2	0.2	78	0	0.2	0	0
Plant-Based Protein Foods	346	22	30	2.5	2.0	0.2	346	22	30	2.5	2.0	0.2	1	0	0.0	0	0
Yogurt	231	23	27	3.1	1.8	0.2	278	23	28	3.1	1.9	0.2	60	1	0.4	0.1	0
Eggs	332	24	27	2.6	1.7	0.2	332	25	24	2.4	1.6	0.2	234	−3	0.5	−0.2	0
Cooked Grains	299	25	26	2.8	1.7	0.2	299	24	26	2.8	1.7	0.2	0	0	0.0	0	0
Mixed Dishes—M/P/F	189	26	22	2.9	1.4	0.2	189	26	21	2.8	1.4	0.2	69	−1	0.2	0	0
Vegetables, excluding Potatoes	560	27	19	1.9	1.2	0.1	560	27	18	1.9	1.2	0.1	22	0	0.2	0	0
Fats and Oils	460	28	17	1.3	1.1	0.1	460	28	17	1.3	1.1	0.1	31	0	0.0	0	0
Mixed Dishes—Soups	227	29	16	2.5	1.0	0.2	227	29	16	2.4	1.0	0.2	7	0	0.1	0	0
Sugars	440	30	15	2.2	1.0	0.1	440	30	16	2.2	1.0	0.1	21	0	0.0	0	0
Meats	220	31	16	2.1	1.0	0.1	220	31	16	2.1	1.0	0.1	5	0	0.0	0	0
**Mean Energy Intake (kcals) in Children 6–11 Years of Age (*n* = 2193)**																	
**WWEIA Food Group**	**Specific Food Group Intake**						**Adjusted Intake**						**Delta Intake**				
**Sub Group Description**	**Cons**	**Rank**	**Mean**	**SE**	**Pct**	**SE**	**Cons**	**Rank**	**Mean**	**SE**	**Pct**	**SE**	**Cons**	**Mean**	**SE**	**Pct**	**SE**
Sweet Bakery Products	1034	1	164	9.1	8.4	0.4	1034	1	163	8.9	8.3	0.4	455	−2	0.2	−0.1	0.01
Mixed Dishes—Pizza	548	2	132	14.6	6.8	0.7	548	6	100	11.1	5.1	0.6	547	−32	3.6	−1.7	0.2
Sweetened Beverages	1467	3	112	4.1	5.7	0.2	1467	3	110	4.1	5.7	0.2	45	−1	0.7	−0.1	0.04
Breads, Rolls, Tortillas	1205	4	109	3.6	5.6	0.2	1205	4	109	3.5	5.6	0.2	85	0	0.1	−0.02	0.00
Mixed Dishes—Grain-based	606	5	105	7.8	5.4	0.4	606	7	95	6.6	4.9	0.3	324	−10	1.5	−0.5	0.1
Mixed Dishes—Mexican	362	6	99	11.0	5.1	0.5	362	9	80	8.8	4.1	0.4	347	−19	2.4	−1.0	0.1
Milk	1274	7	96	3.3	4.9	0.2	1987	2	117	3.4	6.0	0.2	1646	21	1.0	1.0	0.1
Savory Snacks	1048	8	89	4.8	4.6	0.2	1048	8	89	4.8	4.5	0.2	232	0	0.04	−0.01	0.00
Mixed Dishes—Sandwiches	436	9	79	7.0	4.1	0.3	436	11	74	6.6	3.8	0.3	170	−5	0.8	−0.3	0.04
Poultry	711	10	77	5.5	4.0	0.3	711	10	77	5.5	3.9	0.3	124	0	0.03	0.00	0.00
Other Desserts	620	11	65	7.2	3.4	0.4	620	15	56	6.6	2.9	0.3	478	−10	1.0	−0.5	0.1
Ready-to-Eat Cereals	861	12	61	3.7	3.1	0.2	861	12	61	3.7	3.1	0.2	24	0	0.03	0.00	0.00
Quick Breads/Bread Products	475	13	59	4.0	3.0	0.2	475	14	57	3.9	2.9	0.2	445	−3	0.2	−0.1	0.01
Candy	764	14	58	7.4	3.0	0.4	764	13	57	7.3	2.9	0.4	306	−1	0.2	−0.1	0.01
Flavored Milk	555	15	55	5.0	2.8	0.3	555	16	55	5.0	2.8	0.3	0	0	0.00	0.00	0.00
Fruits	1153	16	54	2.4	2.8	0.1	1153	17	54	2.4	2.8	0.1	6	0	0.01	0.00	0.00
White Potatoes	610	17	46	3.3	2.3	0.2	610	18	44	3.2	2.3	0.2	105	−1	0.3	−0.1	0.02
Mixed Dishes—M/F/P	281	18	44	7.4	2.3	0.4	281	19	43	7.2	2.2	0.4	123	−1	0.4	−0.1	0.02
100% Juice	759	19	41	3.2	2.1	0.2	759	20	41	3.2	2.1	0.2	0	0	0.00	0.00	0.00
Cured Meats/Poultry	687	20	38	2.7	1.9	0.1	687	21	38	2.7	1.9	0.1	1	0	0.00	0.00	0.00
Cheese	723	21	38	3.0	1.9	0.2	1643	5	106	4.4	5.4	0.2	1349	69	4.8	3.5	0.2
Plant-Based Protein Foods	475	22	35	3.8	1.8	0.2	475	22	35	3.8	1.8	0.2	1	0	0.00	0.00	0.00
Meats	388	23	31	3.3	1.6	0.2	388	23	31	3.3	1.6	0.2	5	0	0.01	0.00	0.00
Fats and Oils	740	24	29	2.2	1.5	0.1	740	24	29	2.1	1.5	0.1	62	0	0.03	0.00	0.00
Cooked Grains	302	25	27	3.1	1.4	0.2	302	25	27	3.1	1.4	0.2	1	0	0.1	0.00	0.00
Crackers	325	26	25	2.5	1.3	0.1	325	26	25	2.5	1.3	0.1	96	0	0.1	−0.01	0.00
Eggs	324	27	23	2.2	1.2	0.1	324	28	21	2.0	1.1	0.1	217	−2	0.4	−0.1	0.02
Sugars	609	28	21	1.8	1.1	0.1	609	27	21	1.8	1.1	0.1	42	0	0.05	−0.01	0.00
Mixed Dishes—Soups	253	29	20	2.3	1.0	0.1	253	29	19	2.1	1.0	0.1	6	−1	0.5	−0.03	0.02
**Mean Energy Intake (kcals) 12–18 Years of Age (*n* = 2172)**																	
**WWEIA Food Group**	**Specific Food Group Intake**						**Adjusted Intake**						**Delta Intake**				
**Sub Group Description**	**Cons**	**Rank**	**Mean**	**SE**	**Pct**	**SE**	**Cons**	**Rank**	**Mean**	**SE**	**Pct**	**SE**	**Cons**	**Mean**	**SE**	**Pct**	**SE**
Sweetened Beverages	1404	1	162	6.0	7.9	0.3	1404	1	160	5.9	7.8	0.3	40	−1	0.4	−0.1	0.02
Sweet Bakery Products	833	2	139	10.2	6.8	0.5	833	2	137	10.1	6.7	0.5	344	−1	0.2	−0.1	0.01
Mixed Dishes—Pizza	486	3	135	14.2	6.6	0.7	486	6	103	10.8	5.0	0.5	486	−33	3.6	−1.6	0.2
Breads, Rolls, Tortillas	1119	4	118	5.2	5.7	0.2	1119	3	118	5.2	5.7	0.2	85	0.0	0.1	−0.02	0.0
Mixed Dishes—Grain-based	464	5	105	8.0	5.1	0.4	464	7	96	7.6	4.7	0.4	244	−10	1.3	−0.5	0.1
Mixed Dishes—Mexican	329	6	103	11.3	5.0	0.6	329	10	84	9.0	4.1	0.4	313	−19	2.6	−0.9	0.1
Milk	973	7	96	4.8	4.7	0.2	1779	4	117	4.7	5.7	0.2	1453	21.5	1.1	1.0	0.1
Savory Snacks	942	8	93	6.1	4.5	0.3	942	8	92	6.1	4.0	0.3	233	0.0	0.1	−0.02	0.0
Mixed Dishes—Sandwiches	374	9	91	8.6	4.4	0.4	374	11	83	7.6	4.0	0.4	174	−8	1.4	−0.4	0.1
Poultry	652	10	87	8.1	4.2	0.4	652	9	87	8.1	4.2	0.4	130	0.0	0.02	−0.01	0.0
White Potatoes	580	11	63	4.3	3.1	0.2	580	12	60	4.0	2.9	0.2	121	−4	0.7	−0.2	0.03
Ready-to-Eat Cereals	582	12	57	4.0	2.8	0.2	582	13	57	4.0	2.8	0.2	18	0.0	0.01	0.0	0.0
Meats	424	13	54	4.6	2.6	0.2	424	14	54	4.6	2.6	0.2	14	0.0	0.01	0.0	0.0
Mixed Dishes—M/P/F	288	14	49	4.9	2.4	0.2	288	15	46	4.8	2.2	0.2	100	−2	0.6	−0.1	0.03
Other Desserts	377	15	48	5.8	2.3	0.3	377	22	39	4.0	1.9	0.2	339	−9	2.1	−0.5	0.1
Quick Breads/Bread Products	297	16	44	4.1	2.1	0.2	297	17	42	4.0	2.0	0.2	282	−2	0.2	−0.1	0.01
Candy	600	17	43	5.0	2.1	0.2	600	18	42	4.9	2.0	0.2	273	−1	0.2	−0.1	0.01
Cheese	687	18	42	3.1	2.1	0.2	1565	5	118	5.2	5.7	0.3	1276	74	4.9	3.6	0.2
Fruits	834	19	42	3.1	2.1	0.2	834	16	42	3.1	2.1	0.2	3	0.0	0.01	0.0	0.0
Cured Meats/Poultry	585	20	42	4.1	2.0	0.2	585	19	41	4.1	2.0	0.2	2	0.0	0.2	−0.01	0.01
Mixed Dishes—Asian	174	21	41	8.4	2.0	0.4	174	20	41	8.4	2.0	0.4	11	0.0	0.0	0.0	0.0
Plant-Based Protein Foods	344	22	41	6.1	2.0	0.3	344	21	41	6.1	2.0	0.3	1	0.00	0.0	0.0	0.0
Fats and Oils	700	23	38	3.6	1.9	0.2	700	23	38	3.5	1.9	0.2	86	0.0	0.04	−0.01	0.0
100% Juice	487	24	36	4.4	1.8	0.2	487	24	36	4.4	1.8	0.2	0	0.0	0.0	0.0	0.0
Coffee and Tea	505	25	36	5.8	1.8	0.3	505	25	34	5.9	1.6	0.3	60	−3	0.4	−0.1	0.02
Cooked Grains	325	26	31	3.0	1.5	0.2	325	26	31	3.0	1.5	0.2	0	0.0	0.0	0.0	0.0
Eggs	333	27	28	2.3	1.3	0.1	333	28	25	2.1	1.2	0.1	211	−2	0.3	−0.1	0.01
Flavored Milk	225	28	25	3.0	1.2	0.1	225	27	25	3.0	1.2	0.1	0	0.0	0.0	0.0	0.0
Crackers	229	29	25	3.7	1.2	0.2	229	29	25	4.0	1.2	0.2	69	0.0	0.1	−0.01	0.0
Mixed Dishes—Soups	237	30	21	2.0	1.0	0.1	237	31	21	1.9	1.0	0.1	10	−1	0.2	−0.03	0.01
Sugars	446	31	21	3.3	1.0	0.2	446	30	21	3.3	1.0	0.2	16	0.0	0.01	0.0	0.0

^1^ To a 1% contribution of daily intake of energy; ^2^ Nutrients from milk, cheese, and yogurt for non-dairy foods are added to the nutrients in the milk, cheese, and yogurt food categories, respectively. For non-dairy foods the nutrients displayed are only for the milk, cheese, and yogurt in the non-dairy food. Abbreviations: Cons = consumers, M/P/F = meat/poultry/fish; SE = standard error; Pct = percent contribution to energy intake or specific nutrient intake, as appropriate.

**Table 2:** Food/food group sources ^1^ of mean dietary fiber (g) intake among US children aged 2–18 years (*N* = 5876): National Health and Nutrition Examination Survey 2011–2014.

Mean Dietary Fiber Intake (g) of Children 2–5 Years of Age (*n* = 1511)																	
	Specific Food Group Intake						Adjusted Intake ^2^						Delta Intake				
Sub Group Description	Cons	Rank	Mean	SE	Pct	SE	Cons	Rank	Mean	SE	Pct	SE	Cons	Mean	SE	Pct	SE
Fruit	945	1	2.0	0.1	17.2	0.4	945	1	2.0	0.1	17.2	0.4	2	0.0	0.0	0.0	0.0
Breads, Rolls, Tortillas	818	2	1.2	0.1	10.0	0.7	818	2	1.2	0.1	10.0	0.7	30	0.0	0.0	0.0	0.0
Ready-to-Eat Cereals	695	3	0.9	0.1	7.4	0.5	695	3	0.9	0.1	7.4	0.5	12	0.0	0.0	0.0	0.0
Vegetables, excluding Potatoes	560	4	0.9	0.1	7.3	0.5	560	4	0.9	0.1	7.3	0.5	22	0.0	0.0	0.0	0.0
Mixed Dishes—Grain based	466	5	0.8	0.1	7.0	0.7	466	5	0.8	0.1	7.0	0.7	266	0.0	0.0	0.0	0.0
Plant-Based Protein Foods	346	6	0.6	0.1	5.2	0.5	346	6	0.6	0.1	5.2	0.5	1	0.0	0.0	0.0	0.0
Savory Snacks	688	7	0.6	0.0	4.9	0.4	688	7	0.6	0.0	4.9	0.4	140	0.0	0.0	0.0	0.0
Sweet Bakery Products	723	8	0.5	0.0	4.4	0.2	723	8	0.5	0.0	4.4	0.2	262	0.0	0.0	0.0	0.0
Mixed Dishes—Mexican	201	9	0.4	0.1	4.0	0.5	201	9	0.5	0.1	4.0	0.5	189	0.0	0.0	0.0	0.0
Mixed Dishes—Pizza	247	10	0.5	0.1	3.9	0.4	247	10	0.5	0.1	3.9	0.4	247	0.0	0.0	0.0	0.0
White Potatoes	401	11	0.5	0.1	3.8	0.4	401	11	0.5	0.1	3.8	0.4	78	0.0	0.0	0.0	0.0
100% Juice	755	12	0.3	0.0	2.6	0.2	755	12	0.3	0.0	2.6	0.2	0	0.0	0.0	0.0	0.0
Quick Breads and Bread Products	293	13	0.3	0.1	2.5	0.6	293	13	0.3	0.1	2.5	0.6	274	0.0	0.0	0.0	0.0
Flavored Milk	253	14	0.3	0.0	2.2	0.4	253	14	0.3	0.0	2.2	0.4	0	0.0	0.0	0.0	0.0
Crackers	353	15	0.2	0.0	1.9	0.2	353	15	0.2	0.0	1.9	0.2	86	0.0	0.0	0.0	0.0
Poultry	526	16	0.2	0.0	1.5	0.3	526	16	0.2	0.0	1.5	0.3	71	0.0	0.0	0.0	0.0
Cooked Cereal	125	17	0.2	0.0	1.5	0.2	125	17	0.2	0.0	1.5	0.2	69	0.0	0.0	0.0	0.0
Mixed Dishes—Sandwiches	208	18	0.2	0.0	1.5	0.2	208	18	0.2	0.0	1.5	0.2	67	0.0	0.0	0.0	0.0
Mixed Dishes—Soups	227	19	0.2	0.0	1.5	0.3	227	19	0.2	0.0	1.45	0.26	7	0.0	0.0	0.0	0.0
Mixed Dishes—M/P/F	189	20	0.2	0.0	1.4	0.2	189	20	0.2	0.0	1.37	0.22	69	0.0	0.0	0.0	0.0
Cooked Grains	299	21	0.2	0.0	1.4	0.2	299	21	0.2	0.0	1.35	0.23	0	0.0	0.0	0.0	0.0
Snack/Meal Bars	81	22	0.2	0.0	1.3	0.2	81	22	0.2	0.0	1.25	0.19	30	0.0	0.0	0.0	0.0
**Mean Dietary Fiber Intake (g) of Children 6–11 Years of Age (*n* = 2193)**																	
	**Specific Food Group Intake**						**Adjusted Intake**						**Delta Intake**				
**Sub Group Description**	**Cons**	**Rank**	**Mean**	**SE**	**Pct**	**SE**	**Cons**	**Rank**	**Mean**	**SE**	**Pct**	**SE**	**Cons**	**Mean**	**SE**	**Pct**	**SE**
Fruit	1153	1	1.9	0.1	12.8	0.4	1153	1	1.9	0.1	12.8	0.5	6	0.0	0.0	0.0	0.0
Breads, Rolls, Tortillas	1205	2	1.5	0.1	10.0	0.4	1205	2	1.5	0.1	10.0	0.4	85	0.0	0.0	0.0	0.0
Mixed Dishes—Pizza	548	3	1.1	0.1	7.4	0.6	548	3	1.1	0.1	7.4	0.6	547	0.0	0.0	0.0	0.0
Mixed Dishes—Mexican	362	4	1.0	0.1	6.8	0.7	362	4	1.0	0.1	6.8	0.7	347	0.0	0.0	0.0	0.0
Ready-to-Eat Cereals	861	5	1.0	0.1	6.7	0.4	861	5	1.0	0.1	6.7	0.4	24	0.0	0.0	0.0	0.0
Mixed Dishes—Grain based	606	6	1.0	0.1	6.5	0.4	606	6	1.0	0.1	6.5	0.4	324	0.0	0.0	0.0	0.0
Savory Snacks	1048	7	0.9	0.0	6.1	0.3	1048	7	1.0	0.0	6.1	0.3	232	0.0	0.0	0.0	0.0
Vegetables, excluding Potatoes	792	8	0.8	0.1	5.4	0.5	792	8	0.8	0.1	5.4	0.5	40	0.0	0.0	0.0	0.0
Plant-Based Protein Foods	475	9	0.7	0.1	5.0	0.5	475	9	0.7	0.1	5.0	0.5	1	0.0	0.0	0.0	0.0
Sweet Bakery Products	1034	10	0.7	0.0	4.9	0.3	1034	10	0.7	0.0	4.9	0.3	455	0.0	0.0	0.0	0.0
White Potatoes	610	11	0.6	0.0	3.9	0.3	610	11	0.6	0.0	3.9	0.3	105	0.0	0.0	0.0	0.0
Quick Breads and Bread Products	475	12	0.4	0.0	2.5	0.2	475	12	0.4	0.0	2.5	0.2	445	0.0	0.0	0.0	0.0
Mixed Dishes—Sandwiches	436	13	0.3	0.0	2.3	0.2	436	13	0.3	0.0	2.3	0.2	170	0.0	0.0	0.0	0.0
Mixed Dishes—M/P/F	281	14	0.3	0.1	2.2	0.5	281	14	0.3	0.1	2.2	0.5	123	0.0	0.0	0.0	0.0
Flavored Milk	555	15	0.3	0.0	2.1	0.3	555	15	0.3	0.0	2.1	0.3	0	0.0	0.0	0.0	0.0
Poultry	711	16	0.2	0.0	1.6	0.2	711	16	0.2	0.0	1.6	0.2	124	0.0	0.0	0.0	0.0
Other Desserts	620	17	0.2	0.0	1.6	0.2	620	17	0.2	0.0	1.6	0.2	478	0.0	0.0	0.0	0.0
Snack/Meal Bars	124	18	0.2	0.1	1.5	0.3	124	18	0.2	0.1	1.5	0.3	44	0.0	0.0	0.0	0.0
100% Juice	759	19	0.2	0.0	1.4	0.1	759	19	0.2	0.0	1.4	0.1	0	0.0	0.0	0.0	0.0
Mixed Dishes—Soups	253	20	0.2	0.0	1.4	0.2	253	20	0.2	0.0	1.4	0.2	6	0.0	0.0	0.0	0.0
Crackers	325	21	0.2	0.0	1.1	0.2	325	21	0.2	0.0	1.1	0.2	96	0.0	0.0	0.0	0.0
Cooked Grains	302	22	0.2	0.0	1.0	0.1	302	22	0.2	0.0	1.0	0.1	1	0.0	0.0	0.0	0.0
**Mean Dietary Fiber Intake (g) Children 12–18 Years of Age (*n* = 2172)**																	
	**Specific Food Group Intake**						**Adjusted Intake**						**Delta Intake**				
**Sub Group Description**	**Cons**	**Rank**	**Mean**	**SE**	**Pct**	**SE**	**Cons**	**Rank**	**Mean**	**SE**	**Pct**	**SE**	**Cons**	**Mean**	**SE**	**Pct**	**SE**
Fruit	834	1	1.5	0.1	10.0	0.8	834	1	1.5	0.1	10.0	0.8	3	0.0	0.0	0.0	0.0
Bread, Rolls, Tortillas	1119	2	1.5	0.1	10.0	0.6	1119	2	1.5	0.1	10.0	0.6	85	0.0	0.0	0.0	0.0
Mixed Dishes—Mexican	329	3	1.1	0.2	7.7	1.0	329	3	1.1	0.2	7.7	1.0	313	0.0	0.0	0.0	0.0
Mixed Dishes—Pizza	486	4	1.1	0.1	7.4	0.8	486	4	1.1	0.1	7.4	0.8	486	0.0	0.0	0.0	0.0
Mixed Dishes—Grain based	464	5	0.9	0.1	6.4	0.4	464	5	0.9	0.1	6.4	0.4	244	0.0	0.0	0.0	0.0
Vegetables, excluding Potatoes	803	6	0.9	0.1	6.4	0.5	803	6	0.9	0.1	6.4	0.5	28	0.0	0.0	0.0	0.0
Savory Snacks	942	7	0.9	0.1	6.3	0.5	942	7	0.9	0.1	6.3	0.5	233	0.0	0.0	0.0	0.0
Ready-to-Eat Cereals	582	8	0.9	0.1	6.2	0.6	582	8	0.9	0.1	6.2	0.6	18	0.0	0.0	0.0	0.0
Plant-Based Protein Foods	344	9	0.8	0.1	5.7	0.8	344	9	0.8	0.1	5.7	0.8	1	0.0	0.0	0.0	0.0
White Potatoes	580	10	0.8	0.1	5.5	0.4	580	10	0.8	0.1	5.5	0.4	121	0.0	0.0	0.0	0.0
Sweet Bakery Products	833	11	0.6	0.1	4.3	0.3	833	11	0.6	0.1	4.3	0.3	344	0.0	0.0	0.0	0.0
Mixed Dishes—Sandwiches	374	12	0.4	0.0	2.8	0.3	374	12	0.4	0.0	2.8	0.3	174	0.0	0.0	0.0	0.0
Mixed Dishes—M/P/F	288	13	0.3	0.0	2.2	0.2	288	13	0.3	0.0	2.2	0.2	100	0.0	0.0	0.0	0.0
Condiments and Sauces	889	14	0.28	0.03	1.87	0.20	889	14	0.28	0.03	1.9	0.2	41	0.0	0.0	0.0	0.0
Quick Breads and Bread Products	297	15	0.3	0.0	1.9	0.2	297	15	0.3	0.02	1.9	0.2	282	0.0	0.0	0.0	0.0
Poultry	652	16	0.2	0.0	1.6	0.2	652	16	0.2	0.03	1.6	0.2	130	0.0	0.0	0.0	0.0
Snack/Meal Bars	135	17	0.2	0.0	1.6	0.3	135	17	0.2	0.04	1.6	0.3	47	0.0	0.0	0.0	0.0
Mixed Dishes—Soups	237	18	0.2	0.0	1.5	0.2	237	18	0.2	0.03	1.5	0.2	10	0.0	0.0	0.0	0.0
Cooked Grains	325	19	0.2	0.0	1.3	0.1	325	19	0.2	0.02	1.3	0.1	0	0.0	0.0	0.0	0.0
100% Juice	487	20	0.2	0.0	1.3	0.1	487	20	0.2	0.02	1.3	0.1	0	0.0	0.0	0.0	0.0
Mixed Dishes—Asian	174	21	0.2	0.0	1.2	0.2	174	21	0.2	0.03	1.2	0.2	11	0.0	0.0	0.0	0.0
Crackers	229	22	0.2	0.0	1.0	0.2	229	22	0.2	0.02	1.0	0.2	69	0.0	0.0	0.0	0.0

^1^ To a 1% contribution of daily intake of dietary fiber; ^2^ Nutrients from milk, cheese, and yogurt for non-dairy foods are added to the nutrients in the milk, cheese, and yogurt food categories, respectively. For non-dairy foods the nutrients displayed are only for the milk, cheese, and yogurt in the non-dairy food. Abbreviations: Cons = consumers, M/P/F = meat/poultry/fish; SE = standard error; Pct = percent contribution to energy intake or specific nutrient intake, as appropriate.

**Table 3:** Food/food group sources ^1^ of mean calcium (mg) intake among US children aged 2–18 years (*N* = 5876): National Health and Nutrition Examination Survey 2011–2014.

Mean Calcium Intake (mg) of Children 2–5 Years of Age (*n* = 1511)																	
	Specific Food Group Intake						Adjusted Intake ^2^						Delta Intake				
Sub Group Description	Cons	Rank	Mean	SE	Pct	SE	Cons	Rank	Mean	SE	Pct	SE	Cons	Mean	SE	Pct	SE
Milk	1120	1	317.6	12.0	32.7	0.8	1415	1	351.6	11.8	36.2	0.8	1120	34.0	1.7	3.5	0.2
Cheese	542	2	98.9	11.6	10.2	1.0	1047	2	189.5	13.2	19.5	1.1	820	90.6	4.5	9.3	0.4
Flavored Milk	253	3	74.0	8.9	7.6	0.9	253	3	74.0	8.9	7.6	0.9	0	0.0	0.0	0.0	0.0
Breads, Rolls, Tortillas	818	4	43.2	3.2	4.5	0.3	818	5	42.8	3.1	4.4	0.3	30	−0.4	0.3	−0.0	0.0
Yogurt	231	5	41.7	4.7	4.3	0.5	278	4	43.2	4.7	4.5	0.5	60	1.5	0.6	0.2	0.1
100% Juice	755	6	40.4	3.6	4.2	0.4	755	6	40.4	3.6	4.2	0.4	0	0.0	0.0	0.0	0.0
Mixed Dishes—Mexican	201	7	39.7	4.8	4.1	0.5	201	16	9.2	1.1	0.9	0.1	189	−30.6	3.9	−3.1	0.4
Mixed Dishes—Pizza	247	8	35.9	4.1	3.7	0.4	247	33	2.3	0.4	0.2	0.0	247	−33.6	4.0	−3.5	0.4
Mixed Dishes—Grain-based	466	9	27.8	2.2	2.9	0.2	466	14	10.3	1.3	1.1	0.1	266	−17.5	1.7	−1.8	0.2
Ready-to-Eat Cereals	695	10	25.5	1.9	2.6	0.2	695	7	25.4	1.9	2.6	0.2	12	−0.0	0.0	0.0	0.0
Plain Water	1229	11	20.8	1.3	2.1	0.1	1229	8	20.8	1.3	2.1	0.1	0	0.0	0.0	0.0	0.0
Other Desserts	416	12	18.1	2.3	1.9	0.2	416	18	8.0	1.1	0.8	0.1	291	−10.1	1.3	−1.0	0.1
Quick Breads and Bread Products	293	13	18.0	2.4	1.9	0.3	293	10	13.7	1.9	1.4	0.2	274	−4.3	0.8	−0.4	0.1
Dairy Drinks and Substitutes	70	14	16.5	3.4	1.7	0.4	70	9	16.5	3.4	1.7	0.4	0	0.0	0.0	0.0	0.0
Mixed Dishes—Sandwiches	208	15	15.2	1.8	1.6	0.2	208	12	10.5	1.3	1.2	0.1	67	−4.7	0.9	−0.5	0.1
Eggs	332	16	13.0	1.5	1.3	0.2	332	20	6.3	0.6	0.7	0.1	234	−6.7	1.2	−0.7	0.1
Sweet Bakery Products	723	17	12.7	1.2	1.3	0.1	723	13	10.4	0.9	1.1	0.1	262	−2.3	0.3	−0.2	0.0
Fruit	945	18	10.8	0.5	1.1	0.1	945	11	10.8	0.5	1.1	0.1	2	0.0	0.0	0.0	0.0
Vegetables, excluding Potatoes	560	19	10.6	1.0	1.1	0.1	560	15	9.8	1.1	1.0	0.1	22	−0.8	0.5	−0.1	0.1
Cooked Cereals	125	20	10.4	1.5	1.1	0.2	125	22	6.1	0.9	0.6	0.1	69	−4.3	1.0	−0.4	0.1
**Mean Calcium Intake (mg) of Children 6–11 Years of Age (*n* = 2193)**																	
	**Specific Food Group Intake**						**Adjusted Intake**						**Delta Intake**				
**Sub Group Description**	**Cons**	**Rank**	**Mean**	**SE**	**Pct**	**SE**	**Cons**	**Rank**	**Mean**	**SE**	**Pct**	**SE**	**Cons**	**Mean**	**SE**	**Pct**	**SE**
Milk	1274	1	238.2	8.7	22.2	0.7	1987	1	287.3	8.9	26.7	0.8	1646	49.1	2.2	4.6	0.2
Cheese	723	2	107.1	9.5	10.0	0.9	1643	2	281.7	12.3	26.2	0.9	1349	174.6	12.3	16.3	1.0
Mixed Dishes—Pizza	548	3	88.9	9.8	8.3	0.9	548	20	9.5	1.8	0.9	0.2	547	−79.3	9.3	−7.4	0.8
Flavored Milk	555	4	88.3	7.7	8.2	0.7	555	3	88.3	7.7	8.2	0.7	0	0.0	0.0	0.0	0.0
Mixed Dishes—Mexican	362	5	67.9	8.1	6.3	0.8	362	11	16.2	2.0	1.5	0.2	347	−51.7	6.5	−4.8	0.6
Breads, Rolls, Tortillas	1205	6	59.6	3.0	5.6	0.3	1205	4	58.6	2.9	5.5	0.3	85	−1.1	0.2	−0.1	0.0
Mixed Dishes—Grain-based	606	7	39.0	4.5	3.6	0.4	606	14	13.6	1.4	1.3	0.1	324	−25.4	3.7	−2.4	0.3
Other Desserts	620	8	35.1	3.1	3.3	0.3	620	13	14.7	1.3	1.4	0.1	478	−20.4	2.0	−1.9	0.2
100% Juice	759	9	34.8	5.1	3.2	0.5	759	5	34.8	5.1	3.2	0.5	0	0.0	0.0	0.0	0.0
Mixed Dishes—Sandwiches	436	10	31.2	3.6	2.9	0.3	436	10	19.3	2.8	1.8	0.3	170	−12.2	1.7	−1.1	0.2
Quick Breads and Bread Products	475	11	29.6	2.7	2.8	0.3	475	9	23.5	2.5	2.2	0.2	445	−6.1	0.5	−0.6	0.0
Ready-to-Eat Cereals	861	12	28.2	2.4	2.6	0.2	861	6	28.1	2.4	2.6	0.2	24	−0.1	0.1	−0.0	0.0
Plain Water	1732	13	27.9	1.4	2.6	0.1	1732	7	27.9	1.4	2.6	0.1	0	0.0	0.0	0.0	0.0
Yogurt	206	14	22.7	2.6	2.1	0.2	281	8	26.9	2.8	2.5	0.3	93	4.3	1.1	0.4	0.1
Sweet Bakery Products	1034	15	18.9	2.0	1.8	0.2	1034	12	15.3	1.5	1.4	0.2	455	−3.6	0.5	−0.3	0.1
Sweetened Beverages	1467	16	15.3	2.1	1.4	0.2	1467	16	12.7	1.2	1.2	0.1	45	−2.6	1.2	−0.2	0.1
Dairy Drinks and Substitutes	81	17	13.4	2.5	1.2	0.2	81	15	13.4	2.5	1.2	0.2	0	0.0	0.0	0.0	0.0
Fruits	1153	18	11.5	0.6	1.1	0.1	1153	17	11.5	0.5	1.1	0.1	6	−0.0	0.0	0.0	0.0
Vegetables, excluding Potatoes	792	19	10.9	1.0	1.0	0.1	792	18	9.9	1.0	0.9	0.1	40	−1.0	0.3	−0.1	0.0
**Mean Calcium Intake (mg) of Children 12–18 Years of Age (*n* = 2172)**																	
	**Specific Food Group Intake**						**Adjusted Intake**						**Delta Intake**				
**Sub Group Description**	**Cons**	**Rank**	**Mean**	**SE**	**Pct**	**SE**	**Cons**	**Rank**	**Mean**	**SE**	**Pct**	**SE**	**Cons**	**Mean**	**SE**	**Pct**	**SE**
Milk	973	1	239.8	12.5	22.7	1.0	1779	2	289.9	12.6	27.4	0.9	1453	50.1	2.6	4.0	0.2
Cheese	687	2	113.6	5.4	10.8	0.6	1565	1	300.7	12.1	28.5	0.9	1276	187.2	12.1	17.7	0.9
Mixed Dishes—Pizza	486	3	85.6	8.9	8.1	0.8	486	23	6.9	1.0	0.7	0.1	486	−78.7	8.2	−7.5	0.7
Mixed Dishes—Mexican	329	4	68.1	8.7	6.4	0.8	329	10	16.9	2.0	1.6	0.2	313	−51.2	7.1	−4.8	0.6
Breads, Rolls, Tortillas	1119	5	57.3	3.2	5.4	0.3	1119	3	56.1	3.3	5.3	0.3	85	−1.2	0.2	−0.1	0.0
Plain Water	1673	6	53.1	3.1	5.0	0.3	1673	4	53.1	3.1	5.0	0.3	0	0.0	0.0	0.	0.0
Flavored Milk	225	7	40.5	5.1	3.8	0.5	225	5	40.5	5.1	3.8	0.5	0	0.0	0.0	0.0	0.0
Mixed Dishes—Sandwiches	374	8	40.1	4.9	3.8	0.5	374	8	21.5	2.4	2.0	0.2	174	−18.7	3.4	−1.8	0.3
Mixed Dishes—Grain-based	464	9	37.1	3.9	3.5	0.4	464	15	12.1	1.3	1.2	0.1	244	−25.0	3.4	−2.4	0.3
Other Desserts	377	10	30.9	4.4	2.9	0.4	377	16	12.0	1.3	1.1	0.1	339	−18.9	3.3	−1.8	0.3
100% Juice	487	11	28.1	4.0	2.7	0.4	487	6	28.1	4.0	2.7	0.4	0	0.0	0.0	0.0	0.0
Ready-to-Eat Cereals	582	12	27.3	4.4	2.6	0.4	582	7	27.2	4.4	2.6	0.4	18	−0.1	0.0	−0.0	0.0
Quick Breads and Bread Products	297	13	21.3	2.6	2.0	0.2	297	9	17.0	2.3	1.6	0.2	282	−4.2	0.4	−0.4	0.0
Sweet Bakery Products	833	14	16.6	1.4	1.6	0.1	833	13	13.3	1.1	1.3	0.1	344	−3.3	0.4	−0.3	0.0
Sweetened Beverages	1404	15	15.3	1.4	1.5	0.1	1404	14	12.9	1.1	1.2	0.1	40	−2.3	0.6	−0.2	0.1
White Potatoes	580	16	14.6	2.1	1.4	0.2	580	28	4.9	0.4	0.5	0.0	121	−9.7	1.8	−0.9	0.2
Dairy Drinks and Substitutes	79	17	14.5	1.8	1.4	0.2	79	11	14.5	1.8	1.4	0.2	0	0.0	0.0	0.0	0.0
Savory Snacks	942	18	12.5	0.9	1.2	0.1	942	17	11.2	0.8	1.1	0.1	233	−1.3	0.2	−0.1	0.0
Eggs	333	19	12.2	1.1	1.2	0.1	333	24	6.6	0.5	0.6	0.1	211	−5.6	0.7	−0.5	0.1
Vegetables, excluding Potatoes	803	20	12.2	1.2	1.2	0.1	803	18	11.2	1.2	1.1	0.1	28	−1.0	0.3	−0.1	0.0
Mixed Dishes—Meat, Poultry, Fish	288	21	12.1	1.5	1.2	0.1	288	25	6.5	0.7	0.6	0.1	100	−5.7	1.1	−0.5	0.1

^1^ To a 1% contribution of daily intake of calcium; ^2^ Nutrients from milk, cheese, and yogurt for non-dairy foods are added to the nutrients in the milk, cheese, and yogurt food categories, respectively. For non-dairy foods the nutrients displayed are only for the milk, cheese, and yogurt in the non-dairy food. Abbreviations: Cons = consumers, M/P/F = meat/poultry/fish; SE = standard error; Pct = percent contribution to energy intake or specific nutrient intake, as appropriate.

**Table 4:** Food/food group sources ^1^ of mean vitamin D intake (mcg) among US children aged 2–18 years (*N* = 5876): National Health and Nutrition Examination Survey 2011–2014.

Mean Vitamin D Intake (mcg) of Children 2–5 Years of Age (*n* = 1511)																	
WWEIA Food Group	Specific Food Group Intake						Adjusted Intake ^2^						Delta Intake				
Sub Group Description	Cons	Rank	Mean	SE	Pct	SE	Cons	Rank	Mean	SE	Pct	SE	Cons	Mean	SE	Pct	SE
Milk	1120	1	3.3	0.1	52.6	1.5	1415	1	3.5	0.1	55.7	1.5	1120	0.2	0.0	3.0	0.2
Flavored Milk	253	2	0.8	0.1	12.0	1.4	253	2	0.8	0.1	12.0	1.4	0	0.0	0.0	0.0	0.0
Ready-to-Eat Cereals	695	3	0.5	0.0	7.5	0.5	695	3	0.5	0.0	7.5	0.5	12	0.0	0.0	0.0	0.0
Eggs	332	4	0.3	0.0	4.4	0.5	332	5	0.2	0.0	3.8	0.4	234	0.0	0.0	−0.7	0.1
Cheese	542	5	0.3	0.0	4.2	0.4	1047	4	0.3	0.0	5.4	0.5	820	0.1	0.0	1.2	0.1
Seafood	96	6	0.2	0.1	3.2	1.3	96	6	0.2	0.1	3.2	1.3	26	0.0	0.0	0.0	0.0
Yogurt	231	7	0.2	0.0	2.4	0.3	278	7	0.2	0.0	2.4	0.3	60	0.0	0.0	0.0	0.0
100% Juice	755	8	0.1	0.0	1.8	0.3	755	8	0.1	0.0	1.8	0.3	0	0.0	0.0	0.0	0.0
Cured Meats/Poultry	459	9	0.1	0.0	1.7	0.2	459	9	0.1	0.0	1.7	0.2	2	0.0	0.0	0.0	0.0
Dairy Drinks and Substitutes	70	10	0.1	0.0	1.7	0.4	70	10	0.1	0.0	1.7	0.4	0	0.0	0.0	0.0	0.0
Mixed Dishes—Grain-based	466	11	0.1	0.0	1.3	0.1	466	15	0.0	0.0	0.4	0.1	266	−0.1	0.0	−1.0	0.1
**Mean Vitamin D Intake (mcg) of Children 6–11 Years of Age (*n* = 2193)**																	
**WWEIA Food Group**	**Specific Food Group Intake**						**Adjusted Intake**						**Delta Intake**				
**Sub Group Description**	**Cons**	**Rank**	**Mean**	**SE**	**Pct**	**SE**	**Cons**	**Rank**	**Mean**	**SE**	**Pct**	**SE**	**Cons**	**Mean**	**SE**	**Pct**	**SE**
Milk	1274	1	2.4	0.1	42.3	1.1	1987	1	2.7	0.1	46.7	1.1	1646	0.3	0.0	4.5	0.4
Flavored Milk	555	2	0.9	0.1	15.2	1.2	555	2	0.9	0.1	15.2	1.2	0	0.0	0.0	0.0	0.0
Ready-to-Eat Cereals	861	3	0.6	0.0	9.9	0.6	861	3	0.6	0.0	9.9	0.6	24	0.0	0.0	0.0	0.0
Cheese	723	4	0.4	0.0	6.0	0.7	1643	4	0.5	0.0	8.4	0.7	1349	0.1	0.0	2.4	0.2
Eggs	324	5	0.2	0.0	4.1	0.4	324	5	0.2	0.0	3.5	0.4	217	0.0	0.0	−0.6	0.1
Seafood	151	6	0.2	0.0	2.6	0.5	151	6	0.2	0.0	2.6	0.5	47	0.0	0.0	0.0	0.0
100% Juice	759	7	0.1	0.0	2.1	0.4	759	7	0.1	0.0	2.1	0.4	0	0.0	0.0	0.0	0.0
Cured Meats/Poultry	687	8	0.1	0.0	1.9	0.2	687	8	0.1	0.0	1.9	0.2	1	0.0	0.0	0.0	0.0
Mixed Dishes—Sandwiches	436	9	0.1	0.0	1.8	0.2	436	10	0.1	0.0	1.4	0.2	170	0.0	0.0	0.4	0.1
Mixed Dishes—Grain-based	606	10	0.1	0.0	1.7	0.2	606	16	0.0	0.0	0.4	0.1	324	−0.1	0.0	1.3	0.2
Yogurt	206	11	0.1	0.0	1.5	0.2	281	9	0.1	0.0	1.6	0.2	93	0.0	0.0	0.1	0.1
Dairy Drinks and Substitutes	81	12	0.1	0.0	1.3	0.3	81	11	0.1	0.0	1.3	0.3	0	0.0	0.0	0.0	0.0
Mixed Dishes—M/P/F	281	13	0.1	0.0	1.2	0.4	281	13	0.1	0.0	0.8	0.3	123	0.0	0.0	−0.4	0.1
Quick Breads and Bread Products	475	14	0.1	0.0	1.0	0.2	475	15	0.0	0.0	0.5	0.1	445	0.0	0.0	−0.6	0.1
**Mean Vitamin D Intake (mcg) of Children 12–18 Years of Age (*n* = 2172)**																	
**WWEIA Food Group**	**Actual Intake**						**Adjusted Intake**						**Delta Intake**				
**Sub Group Description**	**Cons**	**Rank**	**Mean**	**SE**	**Pct**	**SE**	**Cons**	**Rank**	**Mean**	**SE**	**Pct**	**SE**	**Cons**	**Mean**	**SE**	**Pct**	**SE**
Milk	973	1	2.4	0.1	45.6	1.6	1779	1	2.7	0.1	51.3	1.5	1453	0.3	0.0	5.7	0.4
Ready-to-Eat Cereals	582	2	0.5	0.0	9.2	0.7	582	3	0.5	0.0	9.1	0.7	18	0.0	0.0	0.0	0.0
Flavored Milk	225	3	0.4	0.1	7.6	1.0	225	4	0.4	0.1	7.6	1.0	0	0.0	0.0	0.0	0.0
Cheese	687	4	0.3	0.0	6.4	0.4	1565	2	0.5	0.0	9.6	0.5	1276	0.2	0.0	3.1	0.2
Eggs	333	5	0.3	0.0	5.2	0.4	333	5	0.2	0.0	4.6	0.3	211	0.0	0.0	−0.7	0.2
Seafood	142	6	0.2	0.1	3.7	1.1	142	6	0.2	0.1	3.7	1.1	21	0.00	0.0	0.0	0.0
Cured Meats/Poultry	585	7	0.1	0.0	2.3	0.3	585	7	0.1	0.0	2.3	0.3	2	0.00	0.0	0.0	0.0
Mixed Dishes—Grain-based	464	8	0.1	0.0	2.0	0.3	464	16	0.0	0.0	0.6	0.1	244	−0.1	0.0	−1.5	0.2
Mixed Dishes—Sandwiches	374	9	0.1	0.0	2.0	0.3	374	9	0.1	0.0	1.4	0.2	174	0.0	0.0	−0.6	0.1
100% Juice	487	10	0.1	0.0	1.8	0.3	487	8	0.1	0.0	1.8	0.3	0	0.0	0.0	0.0	0.0
Mixed Dishes—M/P/F	288	11	0.1	0.0	1.7	0.4	288	10	0.1	0.0	1.2	0.4	100	0.0	0.0	−0.5	0.1
White Potatoes	580	12	0.1	0.0	1.2	0.2	580	17	0.0	0.0	0.5	0.1	121	0.0	0.0	−0.7	0.1
Coffee and Tea	505	13	0.1	0.0	1.1	0.2	505	30	0.0	0.0	0.0	0.0	60	−0.1	0.0	−1.0	0.2
Poultry	652	14	0.1	0.0	1.0	0.1	652	11	0.1	0.0	1.0	0.1	130	0.0	0.0	−0.1	0.0

^1^ To a 1% contribution of daily intake of vitamin D; ^2^ Nutrients from milk, cheese, and yogurt for non-dairy foods are added to the nutrients in the milk, cheese, and yogurt food categories, respectively. For non-dairy foods the nutrients displayed are only for the milk, cheese, and yogurt in the non-dairy food. Abbreviations: Cons = consumers, M/P/F = meat/poultry/fish; SE = standard error; Pct = percent contribution to energy intake or specific nutrient intake, as appropriate.

**Table 5:** Food/food group sources ^1^ of mean potassium (mg) intake among US children aged 2–18 years (*N* = 5876): National Health and Nutrition Examination Survey 2011–2014

Mean Potassium Intake (mg) of Children 2–5 Years of Age (*n* = 1511)																	
WWEIA Food Group	Specific Food Group Intake						Adjusted Intake ^2^						Delta Intake				
Sub Group Description	Cons	Rank	Mean	SE	Pct	SE	Cons	Rank	Mean	SE	Pct	SE	Cons	Mean	SE	Pct	SE
Milk	1120	1	375.4	14.4	18.9	0.6	1415	1	417.1	13.9	21.1	0.6	1120	41.8	2.1	2.1	0.1
Fruits	945	2	189.7	7.6	9.6	0.4	945	2	189.7	7.6	9.6	0.4	2	0.00	0.0	0.0	0.0
100% Juice	755	3	169.3	10.0	8.5	0.4	755	3	169.3	10.0	8.5	0.4	0	0.00	0.0	0.0	0.0
Flavored Milk	253	4	100.6	13.1	5.1	0.6	253	4	100.6	13.1	5.1	0.6	0	0.00	0.0	0.0	0.0
Mixed Dishes—Grain-based	466	5	80.1	8.0	4.0	0.4	466	7	72.9	7.9	3.7	0.4	266	−7.2	0.8	−0.4	0.0
Vegetables, excluding Potatoes	560	6	78.5	7.2	4.0	0.4	560	5	78.0	7.3	3.9	0.4	22	−0.5	0.2	0.0	0.0
White Potatoes	401	7	78.3	7.6	4.0	0.4	401	6	76.7	7.5	3.9	0.4	78	−1.6	0.5	−0.1	0.0
Poultry	526	8	69.9	6.6	3.5	0.3	526	8	69.7	6.6	3.5	0.3	71	−0.1	0.0	0.0	0.0
Savory Snacks	688	9	62.5	5.2	3.2	0.3	688	9	62.3	5.2	3.1	0.3	140	−0.2	0.1	0.0	0.0
Sweetened Beverages	787	10	60.9	5.5	3.1	0.3	787	10	59.8	5.4	3.0	0.3	23	−1.1	0.4	−0.1	0.0
Cured Meats/Poultry	459	11	59.4	5.3	3.0	0.3	459	11	59.3	5.3	3.0	0.3	2	−0.1	0.1	0.0	0.0
Yogurt	231	12	53.6	6.0	2.7	0.3	278	12	55.6	5.9	2.8	0.3	60	2.0	0.7	0.1	0.0
Breads, Rolls, Tortillas	818	13	48.9	3.6	2.5	0.2	818	13	48.8	3.6	2.5	0.2	30	−0.1	0.1	0.0	0.0
Plant-Based Protein Foods	346	14	46.8	4.6	2.4	0.2	346	14	46.8	4.6	2.4	0.2	1	0.0	0.0	0.0	0.0
Mixed Dishes—Mexican	201	15	40.3	4.6	2.0	0.2	201	17	35.4	4.1	1.8	0.2	189	−4.9	0.6	−0.3	0.0
Sweet Bakery Products	723	16	39.6	1.6	2.0	0.1	723	15	37.0	1.5	1.9	0.1	262	−2.6	0.4	−0.1	0.0
Mixed Dishes—Pizza	247	17	38.1	4.3	1.9	0.2	247	20	31.8	3.6	1.6	0.2	247	−6.2	0.8	−0.3	0.0
Ready-to-Eat Cereals	695	18	37.0	2.6	1.9	0.1	695	16	36.9	2.6	1.9	0.1	12	−0.1	0.1	0.0	0.0
Mixed Dishes—M/P/F	189	19	33.1	5.6	1.7	0.3	189	19	32.1	5.5	1.6	0.3	69	−1.1	0.3	−0.1	0.0
Other Desserts	416	20	31.3	3.8	1.6	0.2	416	25	18.9	2.4	1.0	0.1	291	−12.4	1.6	−0.6	0.1
Mixed Dishes—Sandwiches	208	21	29.6	3.4	1.5	0.2	208	22	28.6	3.3	1.4	0.2	67	−1.0	0.2	−0.1	0.0
Mixed Dishes—Soups	227	22	29.4	5.9	1.5	0.3	227	21	29.2	5.8	1.5	0.3	7	−0.2	0.1	0.0	0.0
Eggs	332	23	24.3	2.4	1.2	0.1	332	24	20.0	2.0	1.0	0.1	234	−4.2	0.6	−0.2	0.0
Meats	220	24	23.5	2.7	1.2	0.1	220	23	23.5	2.7	1.2	0.1	5	0.0	0.0	0.0	0.0
Quick Bread and Bread Products	293	25	20.5	3.2	1.0	0.2	293	28	15.4	2.4	0.8	0.1	274	−5.1	0.9	−0.3	0.1
**Mean Potassium Intake (mg) of Children 6–11 Years of Age (*n* = 2193)**																	
**WWEIA Food Group**	**Specific Food Group Intake**						**Adjusted Intake**						**Delta Intake**				
**Sub Group Description**	**Cons**	**Rank**	**Mean**	**SE**	**Pct**	**SE**	**Cons**	**Rank**	**Mean**	**SE**	**Pct**	**SE**	**Cons**	**Mean**	**SE**	**Pct**	**SE**
Milk	1274	1	283.4	10.5	12.9	0.5	1987	1	343.5	10.9	15.6	0.5	1646	60.0	2.8	2.7	0.1
Fruits	1153	2	160.3	7.5	7.3	0.3	1153	2	160.3	7.5	7.3	0.3	6	−0.1	0.0	0.0	0.0
Flavored Milk	555	3	124.9	11.2	5.7	0.5	555	3	124.9	11.2	5.7	0.5	0	0.0	0.0	0.0	0.0
100% Juice	759	4	117.5	10.1	5.3	0.4	759	4	117.5	10.1	5.3	0.4	0	0.0	0.0	0.0	0.0
White Potatoes	610	5	101.3	8.0	4.6	0.4	610	5	98.7	7.6	4.5	0.3	105	−2.6	0.7	−0.1	0.0
Mixed Dishes—Pizza	548	6	96.1	9.5	4.4	0.4	548	8	82.1	8.1	3.7	0.4	547	−14.0	1.6	−0.6	0.1
Mixed Dishes—Grain-based	606	7	90.0	6.8	4.1	0.3	606	9	80.7	6.4	3.7	0.3	324	−9.2	1.3	−0.4	0.1
Mixed Dishes—Mexican	362	8	88.0	9.9	4.0	0.4	362	10	79.6	9.1	3.6	0.4	347	−8.4	1.0	−0.4	0.0
Savory Snacks	1048	9	88.0	5.8	4.0	0.3	1048	6	87.8	5.8	4.0	0.3	232	−0.2	0.0	0.0	0.0
Poultry	711	10	85.6	5.4	3.9	0.2	711	7	85.4	5.4	3.9	0.2	124	−0.3	0.1	0.0	0.0
Sweetened Beverages	1467	11	77.6	4.8	3.5	0.2	1467	12	74.4	4.0	3.4	0.2	45	−3.2	1.5	−0.2	0.1
Vegetables, excluding Potatoes	792	12	75.4	6.7	3.4	0.3	792	11	74.8	6.6	3.4	0.3	40	−0.6	0.2	0.0	0.0
Mixed Dishes—M/P/F	281	13	67.1	13.5	3.1	0.6	281	13	64.5	13.2	2.9	0.6	123	−2.6	0.7	−0.1	0.0
Cured Meats/Poultry	687	14	63.0	3.9	2.9	0.2	687	14	62.9	3.9	2.9	0.2	1	0.0	0.0	0.0	0.0
Breads, Rolls, Tortillas	1205	15	62.6	3.5	2.9	0.2	1205	15	62.0	3.4	2.8	0.2	85	−0.6	0.2	0.0	0.0
Mixed Dishes—Sandwiches	436	16	62.3	6.9	2.8	0.3	436	16	59.7	6.8	2.7	0.3	170	−2.6	0.4	−0.1	0.0
Other Desserts	620	17	58.5	4.9	2.7	0.2	620	23	35.1	3.6	1.6	0.2	478	−23.4	1.7	−1.1	0.1
Sweet Bakery Products	1034	18	56.8	3.0	2.6	0.1	1034	17	52.5	2.8	2.4	0.1	455	−4.3	0.6	−0.2	0.0
Plant-Based Protein Foods	475	19	52.0	5.0	2.4	0.2	475	18	52.0	5.0	2.4	0.2	1	0.0	0.0	0.0	0.0
Meats	388	20	47.1	4.7	2.1	0.2	388	20	47.0	4.7	2.1	0.2	5	0.0	0.0	0.0	0.0
Ready-to-Eat Cereals	861	21	38.3	2.3	1.7	0.1	861	21	38.2	2.3	1.7	0.10	24	−0.2	0.1	0.0	0.0
Mixed Dishes—Soups	253	22	38.0	6.3	1.7	0.3	253	22	36.6	5.6	1.7	0.3	6	−1.4	1.4	−0.1	0.1
Condiments and Sauces	867	23	31.5	3.5	1.4	0.2	867	25	30.9	3.4	1.4	0.2	32	−0.6	0.2	0.0	0.0
Yogurt	206	24	29.2	>3.4	1.3	0.2	281	24	34.5	3.6	1.6	0.2	93	5.3	1.4	0.2	0.1
Quick Breads and Bread Products	475	25	27.9	1.5	1.2	0.1	475	26	20.7	1.2	0.9	0.1	445	−7.3	0.6	−0.3	0.0
**Mean Potassium Intake (mg) of Children 12–18 Years of Age (*n* = 2172)**																	
**WWEIA Food Group**	**Specific Food Group Intake**						**Adjusted Intake**						**Delta Intake**				
**Sub Group Description**	**Cons**	**Rank**	**Mean**	**SE**	**Pct**	**SE**	**Cons**	**Rank**	**Mean**	**SE**	**Pct**	**SE**	**Cons**	**Mean**	**SE**	**Pct**	**SE**
Milk	973	1	285.4	15.1	12.4	0.6	1779	1	346.7	15.3	15.0	0.7	1453	61.3	3.2	2.7	0.2
White Potatoes	580	2	145.2	11.5	6.3	0.4	580	2	140.9	10.9	6.1	0.4	121	−4.3	0.8	−0.2	0.0
Fruits	834	3	128.8	9.7	5.6	0.4	834	3	128.8	9.7	5.6	0.4	3	0.0	0.0	0.0	0.0
100% Juice	487	4	107.1	9.8	4.6	0.4	487	4	107.1	9.8	4.6	0.4	0	0.0	0.0	0.0	0.
Poultry	652	5	103.8	10.1	4.5	0.4	652	5	103.5	10.0	4.5	0.4	130	−0.3	0.1	0.0	0.00
Mixed Dishes—Mexican	329	6	97.7	10.9	4.2	0.5	329	8	89.4	10.0	3.9	0.4	313	−8.3	1.1	0.4	0.1
Mixed Dishes—Pizza	486	7	96.5	10.0	4.2	0.4	486	11	82.3	8.5	3.6	0.4	486	−14.2	1.5	−0.6	0.1
Mixed Dishes—Grain-based	464	8	96.1	7.9	4.2	0.3	464	9	86.4	7.5	3.7	0.3	244	−9.7	1.	−0.4	0.1
Vegetables, excluding Potatoes	803	9	95.4	8.6	4.1	0.3	803	6	94.8	8.6	4.1	0.3	28	−0.6	0.23	0.0	0.0
Savory Snacks	942	10	92.5	7.1	4.0	0.3	942	7	92.2	7.1	4.0	0.3	233	−0.3	0.1	0.0	0.0
Meats	424	11	85.8	7.7	3.7	0.3	424	10	85.7	7.7	3.7	0.3	14	−0.1	0.0	0.0	0.0
Mixed Dishes—Sandwiches	374	12	74.6	7.5	3.2	0.3	374	13	70.8	6.9	3.1	0.3	174	−3.9	0.6	−0.2	0.0
Cured Meats/Poultry	585	13	72.1	5.7	3.1	0.2	585	12	72.0	5.7	3.1	0.2	2	−0.1	0.1	0.0	0.0
Mixed Dishes—M/P/F	288	14	71.2	9.3	3.1	0.4	288	14	67.8	9.3	2.9	0.4	100	−3.4	0.7	−0.2	0.0
Sweetened Beverages	1404	15	67.1	5.4	2.9	0.3	1404	15	64.0	4.8	2.8	0.2	40	−3.0	0.8	−0.1	0.0
Breads, Rolls, Tortillas	1119	16	63.7	3.4	2.8	0.1	1119	16	63.0	3.4	2.7	0.1	85	−0.7	0.1	0.0	0.0
Plant-Based Protein Foods	344	17	59.5	9.8	2.6	0.4	344	17	59.5	9.8	2.6	0.4	1	0.0	0.0	0.0	0.0
Flavored Milk	225	18	58.3	7.1	2.5	0.3	225	18	58.3	7.1	2.5	0.3	0	0.0	0.0	0.0	0.0
Coffee and Tea	505	19	52.2	3.4	2.3	0.2	505	23	44.9	3.6	1.9	0.2	60	−7.3	1.2	−0.3	0.1
Sweet Bakery Products	833	20	50.7	4.4	2.2	0.2	833	20	46.9	4.1	2.0	0.2	344	−3.8	0.5	−0.2	0.0
Other Desserts	377	21	48.9	6.1	2.1	0.3	377	26	26.1	2.7	1.1	0.1	339	−22.7	3.8	−1.0	0.2
Condiments and Sauces	889	22	47.1	4.6	2.0	0.2	889	21	46.1	4.6	2.0	0.2	41	−1.0	0.4	0.0	0.0
Mixed Dishes—Asian	174	23	45.0	10.8	2.0	0.5	174	22	45.0	10.8	2.0	0.5	11	0.0	0.0	0.0	0.0
Ready-to-Eat Cereals	582	24	39.1	3.7	1.7	0.2	582	24	39.0	3.7	1.7	0.2	18	−0.1	0.04	0.00	0.0
Mixed Dishes—Soups	237	25	30.6	4.2	1.3	0.2	237	25	29.7	4.0	1.3	0.2	10	−0.9	0.35	0.0	0.0
Eggs	333	26	24.9	2.0	1.1	0.1	333	27	21.2	1.7	0.9	0.1	211	−3.8	0.38	−0.2	0.0

^1^ To a 1% contribution of daily intake of potassium; ^2^ Nutrients from milk, cheese, and yogurt for non-dairy foods are added to the nutrients in the milk, cheese, and yogurt food categories, respectively. For non-dairy foods the nutrients displayed are only for the milk, cheese, and yogurt in the non-dairy food. Abbreviations: Cons = consumers, M/P/F = meat/poultry/fish; SE = standard error; Pct = percent contribution to energy intake or specific nutrient intake, as appropriate.

**Table 6:** Food/food group sources ^1^ of mean added sugars (tsp eq) intake among US children aged 2–18 years (*N* = 5876): National Health and Nutrition Examination Survey 2011–2014.

Mean Added Sugars Intake (tsp eq) of Children 2–5 Years of Age (*n* = 1511)																	
	Specific Food Group Intake						Adjusted Intake ^2^						Delta Intake				
Sub Group Description	Cons	Rank	Mean	SE	Pct	SE	Cons	Rank	Mean	SE	Pct	SE	Cons	Mean	SE	Pct	SE
Sweetened Beverages	787	1	3.0	0.2	25.3	1.4	787	1	3.0	0.2	25.2	1.4	23	0.0	0.0	−0.1	0.0
Sweet Bakery Products	723	2	1.9	0.1	16.0	0.7	723	2	1.9	0.1	16.0	0.7	262	0.0	0.0	0.0	0.0
Other Desserts	416	3	0.9	0.1	7.5	0.9	416	3	0.9	0.1	7.4	0.9	291	0.0	0.0	−0.1	0.1
Ready-to-Eat Cereals	695	4	0.9	0.1	7.2	0.5	695	4	0.9	0.1	7.2	0.5	12	0.0	0.0	0.0	0.0
Candy	507	5	0.8	0.1	6.9	0.4	507	5	0.8	0.1	6.9	0.4	181	0.0	0.0	0.0	0.0
Flavored Milk	253	6	0.8	0.1	6.7	0.8	253	6	0.8	0.1	6.7	0.8	0	0.0	0.0	0.0	0.0
Sugars	440	7	0.7	0.1	6.2	1.0	440	7	0.7	0.1	6.2	1.0	21	0.0	0.0	0.0	0.0
Yogurt	231	8	0.6	0.1	5.1	0.7	278	8	0.6	0.1	5.3	0.7	60	0.0	0.0	0.2	0.2
Quick Breads and Bread Products	293	9	0.4	0.1	3.0	0.7	293	9	0.4	0.1	3.0	0.7	274	0.0	0.0	0.0	0.0
Breads, Rolls, Tortillas	818	10	0.3	0.0	2.1	0.2	818	10	0.3	0.0	2.1	0.2	30	0.0	0.0	0.0	0.0
Dairy Drinks and Substitutes	70	11	0.2	0.0	1.5	0.3	70	11	0.2	0.0	1.5	0.3	0	0.0	0.0	0.0	0.0
Fruit	945	12	0.2	0.0	1.5	0.2	945	12	0.2	0.0	1.5	0.2	2	0.0	0.0	0.0	0.0
Coffee and Tea	130	13	0.2	0.0	1.5	0.2	130	13	0.2	0.0	1.5	0.2	6	0.0	0.0	0.0	0.0
Snack/Meal Bars	81	14	0.2	0.0	1.4	0.2	81	14	0.2	0.0	1.4	0.2	30	0.0	0.0	0.0	0.0
Condiments and Sauces	487	15	0.2	0.0	1.3	0.1	487	15	0.2	0.0	1.3	0.1	18	0.0	0.0	0.0	0.0
**Mean Added Sugars Intake (tsp eq) of Children 6–11 Years of Age (*n* = 2193)**																	
	**Specific Food Group Intake**						**Adjusted Intake ^2^**						**Delta Intake**				
**Sub Group Description**	**Cons**	**Rank**	**Mean**	**SE**	**Pct**	**SE**	**Cons**	**Rank**	**Mean**	**SE**	**Pct**	**SE**	**Cons**	**Mean**	**SE**	**Pct**	**SE**
Sweetened Beverages	1467	1	5.8	0.2	32.1	1.06	1467	1	5.82	0.2	31.7	1.1	45	0.0	0.0	−0.1	0.1
Sweet Bakery Products	1034	2	2.8	0.2	15.3	0.8	1034	2	2.8	0.2	15.3	0.8	455	0.0	0.0	0.0	0.0
Candy	764	3	1.5	0.2	8.2	0.9	764	3	1.5	0.2	8.2	0.9	306	0.0	0.0	0.0	0.0
Other Desserts	620	4	1.4	0.2	7.8	0.8	620	4	1.4	0.2	7.6	0.8	478	0.0	0.0	−0.2	0.1
Ready-to-Eat Cereals	861	5	1.1	0.1	6.2	0.5	861	5	1.1	0.1	6.2	0.5	24	0.0	0.0	0.0	0.00
Sugars	609	6	0.9	0.1	5.1	0.4	609	6	0.9	0.1	5.1	0.4	42	0.0	0.0	0.0	0.0
Flavored Milk	555	7	0.9	0.1	4.9	0.4	555	7	0.9	0.1	4.9	0.4	0	0.0	0.0	0.0	0.0
Coffee and Tea	285	8	0.7	0.1	3.8	0.6	285	8	0.7	0.1	3.8	0.6	16	0.0	0.0	0.0	0.0
Quick Breads and Bread Products	475	9	0.5	0.0	2.6	0.2	475	9	0.5	0.0	2.6	0.2	445	0.0	0.0	0.0	0.0
Breads, Rolls, Tortillas	1205	10	0.4	0.0	2.0	0.1	1205	11	0.4	0.0	2.0	0.1	85	0.0	0.0	0.0	0.0
Yogurt	206	11	0.3	0.0	1.7	0.2	281	10	0.4	0.0	2.1	0.2	93	0.1	0.0	0.4	0.1
Condiments and Sauces	867	12	0.3	0.0	1.4	0.2	867	12	0.3	0.0	1.4	0.2	32	0.0	0.0	0.0	0.0
Dairy Drinks and Substitutes	81	13	0.2	0.1	1.3	0.3	81	13	0.2	0.0	1.3	0.3	0	0.0	0.0	0.0	0.0
Mixed Dishes Sandwiches	436	14	0.2	0.0	1.2	0.1	436	14	0.2	0.0	1.2	0.1	170	0.0	0.0	0.0	0.0
**Mean Added Sugars Intake (tsp eq) of Children 12–18 Years of Age (*n* = 2172)**																	
	**Specific Food Group Intake**						**Adjusted Intake**						**Delta Intake**				
**Sub Group Description**	**Cons**	**Rank**	**Mean**	**SE**	**Pct**	**SE**	**Cons**	**Rank**	**Mean**	**SE**	**Pct**	**SE**	**Cons**	**Mean**	**SE**	**Pct**	**SE**
Sweetened Beverages	1404	1	8.6	0.3	42.5	1.3	1404	1	8.6	0.3	42.4	1.3	40	0.0	0.0	−0.1	0.0
Sweet Bakery Products	833	2	2.4	0.2	11.8	0.8	833	2	2.4	0.2	11.8	0.8	344	0.0	0.0	0.0	0.0
Coffee and Tea	505	3	1.7	0.3	8.3	1.4	505	3	1.7	0.3	8.3	1.4	60	0.0	0.0	0.0	0.0
Candy	600	4	1.1	0.2	5.6	0.7	600	4	1.1	0.2	5.6	0.7	273	0.0	0.0	0.0	0.0
Ready-to-Eat Cereals	582	5	1.0	0.1	5.0	0.4	582	5	1.0	0.1	5.0	0.4	18	0.0	0.0	0.0	0.0
Sugars	446	6	1.0	0.1	5.0	0.7	446	6	1.0	0.1	4.9	0.7	16	0.0	0.0	0.0	0.0
Other Desserts	377	7	1.0	0.1	4.9	0.5	377	7	0.9	0.1	4.6	0.4	339	−0.1	0.0	−0.3	0.2
Flavored Milk	225	8	0.5	0.1	2.2	0.3	225	8	0.5	0.1	2.2	0.3	0	0.0	0.0	0.0	0.0
Breads, Rolls, Tortillas	1119	9	0.4	0.0	1.8	0.1	1119	9	0.4	0.0	1.8	0.1	85	0.0	0.0	0.0	0.0
Quick Breads and Bread Products	297	10	0.4	0.1	1.7	0.2	297	10	0.4	0.1	1.7	0.2	282	0.0	0.0	0.0	0.0
Dairy Drinks and Substitutes	79	11	0.3	0.1	1.4	0.3	79	11	0.3	0.1	1.4	0.3	0	0.0	0.0	0.0	0.0
Snack/Meal Bars	135	12	0.3	0.0	1.3	0.2	135	12	0.3	0.0	1.3	0.2	47	0.0	0.0	0.0	0.0
Condiments and Sauces	889	13	0.2	0.0	1.2	0.1	889	13	0.2	0.0	1.2	0.1	41	0.0	0.0	0.0	0.0

^1^ To a 1% contribution of daily intake of added sugars; ^2^ Nutrients from milk, cheese, and yogurt for non-dairy foods are added to the nutrients in the milk, cheese, and yogurt food categories, respectively. For non-dairy foods the nutrients displayed are only for the milk, cheese, and yogurt in the non-dairy food. Abbreviations: Cons = consumers, M/P/F = meat/poultry/fish; SE = standard error; Pct = percent contribution to energy intake or specific nutrient intake, as appropriate.

**Table 7:** Food/food group sources ^1^ of mean saturated fatty acids (g) intake among US children aged 2–18 years (*N* = 5876): National Health and Nutrition Examination Survey 2011–2014.

Mean Saturated Fatty Acids Intake (g) of Children 2 to 5 Years of Age (*n* = 1511)																	
WWEIA Food Group	Specific Food Group Intake						Adjusted Intake ^2^						Delta Intake				
Sub Group Description	Cons	Rank	Mean	SE	Pct	SE	Cons	Rank	Mean	SE	Pct	SE	Cons	Mean	SE	Pct	SE
Milk	1120	1	3.4	0.2	16.7	0.6	1415	1	3.7	0.2	18.4	0.6	1120	0.3	0.0	1.6	0.1
Sweet Bakery Products	723	2	1.8	0.1	8.8	0.5	723	3	1.8	0.1	8.7	0.5	262	0.0	0.0	−0.1	0.0
Cheese	542	3	1.7	0.2	8.2	1.0	1047	2	3.3	0.3	16.4	1.0	820	1.6	0.1	8.1	0.4
Mixed Dishes—Grain-based	466	4	1.2	0.1	5.7	0.5	466	5	0.9	0.1	4.3	0.4	266	−0.3	0.0	−1.4	0.1
Mixed Dishes—Mexican	201	5	1.1	0.1	5.7	0.6	201	10	0.6	0.1	3.1	0.3	189	−0.5	0.1	−2.6	0.3
Cured Meats/Poultry	459	6	1.0	0.1	4.8	0.5	459	4	1.0	0.1	4.8	0.5	2	0.0	0.0	0.0	0.0
Mixed Dishes—Pizza	247	7	0.9	0.1	4.7	0.6	247	21	0.3	0.0	1.4	0.2	247	−0.7	0.1	−3.3	0.4
Flavored Milk	253	8	0.8	0.1	3.9	0.5	253	6	0.8	0.1	3.9	0.5	0	0.0	0.0	0.0	0.0
Other Desserts	416	9	0.8	0.1	3.9	0.5	416	7	0.7	0.1	3.4	0.4	291	−0.1	0.0	−0.5	0.1
Mixed Dishes—Sandwiches	208	10	0.7	0.1	3.4	0.3	208	11	0.6	0.1	3.1	0.3	67	−0.1	0.0	−0.4	0.1
Eggs	332	11	0.7	0.1	3.3	0.4	332	12	0.6	0.1	2.8	0.3	234	−0.1	0.0	−0.5	0.1
Fats and Oils	460	12	0.7	0.1	3.2	0.3	460	8	0.7	0.1	3.2	0.3	31	0.0	0.0	0.	0.0
Poultry	526	13	0.7	0.1	3.2	0.3	526	9	0.7	0.1	3.2	0.3	71	0.0	0.0	0.0	0.0
Savory Snacks	688	14	0.5	0.0	2.6	0.2	688	13	0.5	0.0	2.6	0.2	140	0.0	0.0	−0.1	0.0
Candy	507	15	0.5	0.1	2.4	0.3	507	14	0.5	0.1	2.3	0.3	181	0.0	0.0	−0.1	0.0
Crackers	353	16	0.4	0.0	1.9	0.2	353	16	0.4	0.0	1.8	0.2	86	0.0	0.0	−0.1	0.0
Plant-Based Protein Foods	346	17	0.4	0.0	1.9	0.2	346	15	0.4	0.0	1.9	0.2	1	0.0	0.0	0.0	0.0
Quick Breads and Bread Products	293	18	0.4	0.1	1.8	0.3	293	18	0.3	0.0	1.6	0.2	274	0.0	0.0	−0.2	0.0
Breads, Rolls, Tortillas	818	19	0.4	0.1	1.7	0.2	818	17	0.3	0.1	1.7	0.2	30	0.0	0.0	0.0	0.0
Meats	220	20	0.3	0.1	1.5	0.3	220	19	0.3	0.1	1.5	0.3	5	0.0	0.0	0.0	0.0
White Potatoes	401	21	0.3	0.0	1.4	0.2	401	22	0.3	0.0	1.3	0.2	78	0.0	0.0	−0.1	0.0
Mixed Dishes—M/P/F	189	22	0.3	0.0	1.4	0.2	189	23	0.3	0.0	1.3	0.1	69	0.0	0.0	−0.1	0.0
Yogurt	231	23	0.3	0.0	1.3	0.2	278	20	0.3	0.0	1.4	0.2	60	0.0	0.0	0.1	0.0
**Mean Intake of Saturated Fatty Acids Intake (g) of Children 6–11 Years of Age (*n* = 2193)**																	
**WWEIA Food Group**	**Specific Food Group Intake**						**Adjusted Intake**						**Delta Intake**				
**Sub Group Description**	**Cons**	**Rank**	**Mean**	**SE**	**Pct**	**SE**	**Cons**	**Rank**	**Mean**	**SE**	**Pct**	**SE**	**Cons**	**Mean**	**SE**	**Pct**	**SE**
Sweet Bakery Products	1034	1	2.6	0.2	10.0	0.7	1034	3	2.6	0.2	9.9	0.7	455	0.0	0.0	−0.1	0.0
Mixed Dishes—Pizza	548	2	2.3	0.3	9.0	1.0	548	11	0.8	0.1	3.2	0.4	547	−1.5	0.2	−5.7	0.6
Milk	1274	3	2.2	0.1	8.3	0.4	1987	2	2.6	0.1	10.1	0.4	1646	0.5	0.0	1.8	0.1
Mixed Dishes—Mexican	362	4	2.0	0.2	7.9	0.9	362	6	1.2	0.1	4.5	0.5	347	−0.9	0.1	−3.4	0.4
Cheese	723	5	1.6	0.1	6.1	0.5	1643	1	4.7	0.2	18.3	0.7	1349	3.2	0.2	12.1	0.7
Other Desserts	620	6	1.5	0.1	5.9	0.5	620	4	1.4	0.1	5.2	0.5	478	−0.2	0.0	−0.7	0.1
Mixed Dishes—Grain-based	606	7	1.5	0.2	5.7	0.8	606	7	1.1	0.2	4.1	0.6	324	−0.4	0.1	−1.6	0.2
Mixed Dishes—Sandwiches	436	8	1.4	0.1	5.5	0.5	436	5	1.2	0.1	4.6	0.4	170	−0.2	0.0	−0.9	0.1
Fats and Oils	740	9	1.0	0.1	4.0	0.4	740	8	1.0	0.1	4.0	0.4	62	0.0	0.0	0.0	0.0
Poultry	711	10	0.9	0.1	3.4	0.3	711	9	0.9	0.1	3.4	0.3	124	0.0	0.0	0.0	0.0
Cured Meats/Poultry	687	11	0.9	0.1	3.3	0.3	687	10	0.9	0.1	3.3	0.3	1	0.0	0.0	0.0	0.0
Savory Snacks	1048	12	0.8	0.1	3.3	0.2	1048	12	0.8	0.1	3.2	0.2	232	0.0	0.0	−0.1	0.0
Candy	764	13	0.8	0.2	3.2	0.8	764	13	0.8	0.2	3.1	0.8	306	0.0	0.0	−0.1	0.0
Flavored Milk	555	14	0.8	0.1	2.9	0.3	555	14	0.8	0.1	2.9	0.3	0	0.0	0.0	0.0	0.0
Meats	388	15	0.6	0.1	2.4	0.3	388	15	0.6	0.1	2.4	0.3	5	0.0	0.0	0.0	0.0
Mixed Dishes—M/P/F	281	16	0.6	0.1	2.3	0.4	281	16	0.6	0.1	2.1	0.4	123	−0.1	0.0	−0.2	0.1
Eggs	324	17	0.6	0.1	2.2	0.2	324	17	0.5	0.1	1.9	0.2	217	−0.1	0.0	−0.3	0.1
Quick Breads and Bread Products	475	18	0.5	0.1	2.0	0.2	475	18	0.5	0.1	1.8	0.2	445	−0.1	0.0	−0.2	0.0
White Potatoes	610	19	0.4	0.0	1.6	0.1	610	21	0.4	0.0	1.5	0.1	105	0.0	0.0	−0.1	0.0
Plant-Based Protein Foods	475	20	0.4	0.1	1.6	0.2	475	19	0.4	0.1	1.6	0.2	1	0.0	0.0	0.0	0.0
Breads, Rolls, Tortillas	1205	21	0.4	0.0	1.6	0.1	1205	20	0.4	0.0	1.5	0.1	85	0.0	0.0	−0.1	0.0
**Mean Saturated Fatty Acids (g) Intake of Children 12–18 Years of Age (*n* = 2172)**																	
**WWEIA Food Group**	**Specific Food Group Intake**						**Adjusted Intake**						**Delta Intake**				
**Sub Group Description**	**Cons**	**Rank**	**Mean**	**SE**	**Pct**	**SE**	**Cons**	**Rank**	**Mean**	**SE**	**Pct**	**SE**	**Cons**	**Mean**	**SE**	**Pct**	**SE**
Mixed Dishes—Pizza	486	1	2.4	0.2	9.1	0.9	486	12	0.9	0.1	3.3	0.3	486	−1.5	0.2	−5.8	0.6
Sweet Bakery Products	833	2	2.2	0.2	8.6	0.6	833	3	2.2	0.2	8.5	0.6	344	0.0	0.0	−0.1	0.0
Milk	973	3	2.1	0.1	8.1	0.5	1779	2	2.6	0.1	10.0	0.4	1453	0.5	0.0	1.9	0.1
Mixed Dishes—Mexican	329	4	2.1	0.2	7.9	0.9	329	5	1.2	0.1	4.5	0.5	313	−0.9	0.1	−3.4	0.5
Cheese	687	5	1.7	0.1	6.7	0.4	1565	1	5.1	0.2	19.7	0.7	1276	3.4	0.2	13.1	0.7
Mixed Dishes—Sandwiches	374	6	1.6	0.2	6.1	0.7	374	4	1.3	0.1	4.8	0.5	174	−0.3	0.1	−1.3	0.2
Mixed Dishes—Grain-based	464	7	1.4	0.1	5.5	0.5	464	8	1.0	0.1	3.9	0.4	244	−0.4	0.1	−1.6	0.2
Other Desserts	377	8	1.2	0.1	4.6	0.5	377	7	1.0	0.1	3.9	0.4	339	−0.2	0.0	−0.6	0.1
Meats	424	9	1.0	0.1	4.0	0.4	424	6	1.0	0.1	4.0	0.4	14	0.0	0.0	0.0	0.0
Poultry	652	10	1.0	0.1	3.8	0.4	652	9	1.0	0.1	3.8	0.4	130	0.0	0.0	0.0	0.0
Fats and Oils	700	11	1.0	0.1	3.8	0.4	700	10	1.0	0.1	3.8	0.4	86	0.0	0.0	0.0	0.0
Cured Meats/Poultry	585	12	0.9	0.1	3.6	0.4	585	11	0.9	0.1	3.5	0.4	2	0.0	0.0	0.0	0.0
Savory Snacks	942	13	0.8	0.1	3.2	0.4	942	13	0.8	0.1	3.1	0.4	233	0.0	0.0	−0.1	0.0
Candy	600	14	0.7	0.1	2.6	0.3	600	14	0.6	0.1	2.5	0.3	273	0.0	0.0	−0.1	0.0
Mixed Dishes—M/P/F	288	15	0.7	0.1	2.6	0.3	288	16	0.6	0.1	2.2	0.3	100	−0.1	0.0	−0.4	0.1
Eggs	333	16	0.7	0.1	2.5	0.2	333	15	0.6	0.1	2.2	0.2	211	−0.1	0.0	−0.3	0.0
White Potatoes	580	17	0.6	0.1	2.2	0.2	580	19	0.5	0.0	1.7	0.2	121	−0.1	0.0	−0.5	0.1
Breads, Rolls, Tortillas	1119	18	0.5	0.0	2.0	0.1	1119	17	0.5	0.0	1.9	0.1	85	0.0	0.0	−0.1	0.0
Plant-Based Protein Foods	344	19	0.5	0.1	1.8	0.3	344	18	0.5	0.1	1.8	0.3	1	0.0	0.0	0.0	0.0
Quick Breads and Bread Products	297	20	0.4	0.1	1.6	0.2	297	20	0.4	0.0	1.4	0.2	282	0.0	0.0	−0.2	0.0
Dairy Drinks and Substitutes	79	21	0.4	0.1	1.3	0.2	79	21	0.4	0.1	1.3	0.2	0	0.0	0.0	0.0	0.0
Flavored Milk	225	22	0.3	0.1	1.3	0.2	225	22	0.3	0.1	1.3	0.2	0	0.0	0.0	0.0	0.0
Mixed Dishes—Asian	174	23	0.3	0.1	1.2	0.3	174	23	0.3	0.1	1.2	0.3	11	0.0	0.0	0.0	0.0
Mixed Dishes—Soups	237	24	0.3	0.0	1.1	0.1	237	24	0.3	0.0	1.0	0.1	10	0.0	0.0	−0.1	0.0

^1^ To a 1% contribution of daily intake of SFA; ^2^ Nutrients from milk, cheese, and yogurt for non-dairy foods are added to the nutrients in the milk, cheese, and yogurt food categories, respectively. For non-dairy foods the nutrients displayed are only for the milk, cheese, and yogurt in the non-dairy food. Abbreviations: Cons = consumers, M/P/F = meat/poultry/fish; SE = standard error; Pct = percent contribution to energy intake or specific nutrient intake, as appropriate.

**Table 8:** Food/food group sources ^1^ of mean sodium (mg) intake among US children aged 2–18 years (*N* = 5876): National Health and Nutrition Examination Survey 2011–2014.

Mean Sodium Intake (mg) of Children 2–5 Years of Age (*n* = 1511)																	
WWEIA Food Group	Actual Intake						Adjusted Intake ^2^						Delta Intake				
Sub Group Description	Cons	Rank	Mean	SE	Pct	SE	Cons	Rank	Mean	SE	Pct	SE	Cons	Mean	SE	Pct	SE
Cured Meats/Poultry	459	1	182.7	20.3	8.1	0.8	459	2	182.4	20.2	8.0	0.8	2	−0.3	0.3	0.0	0.0
Mixed Dishes—Grain-based	466	2	155.1	12.3	6.8	0.5	466	4	140.3	11.8	6.2	0.5	266	−14.9	1.4	−0.7	0.1
Breads, Rolls, Tortillas	818	3	144.4	10.6	6.4	0.5	818	3	144.0	10.5	6.4	0.5	30	−0.4	0.2	0.0	0.0
Poultry	526	4	131.3	11.4	5.8	0.5	526	6	131.2	11.4	5.8	0.5	71	0.0	0.0	0.0	0.0
Milk	1120	5	120.7	4.4	5.3	0.2	1415	5	133.8	4.2	5.9	0.2	1120	13.2	0.7	0.6	0.0
Mixed Dishes—Pizza	247	6	118.4	13.4	5.2	0.6	247	11	83.1	9.4	3.7	0.4	247	−35.3	4.3	−1.6	0.2
Mixed Dishes—Mexican	201	7	116.4	13.3	5.1	0.6	201	9	88.7	10.2	3.9	0.5	189	−27.7	3.5	−1.2	0.2
Cheese	542	8	99.5	11.6	4.4	0.5	1047	1	187.5	13.1	8.3	0.5	820	88.0	4.4	3.9	0.2
Mixed Dishes—Sandwiches	208	9	97.2	10.1	4.3	0.4	208	7	92.9	9.7	4.1	0.4	67	−4.3	0.9	−0.2	0.0
Sweet Bakery Products	723	10	90.6	3.8	4.0	0.2	723	8	89.7	3.8	4.0	0.2	262	−0.9	0.1	0.0	0.0
Savory Snacks	688	11	85.8	11.6	3.8	0.5	688	10	85.3	11.6	3.8	0.5	140	−0.5	0.1	0.0	0.0
Quick Breads and Bread Products	293	12	74.2	8.3	3.3	0.4	293	13	72.6	8.1	3.2	0.4	274	−1.6	0.3	−0.1	0.0
Mixed Dishes—Soups	227	13	72.9	10.8	3.2	0.5	227	12	72.7	10.8	3.2	0.5	7	−0.2	0.1	0.0	0.0
Ready-to-Eat Cereals	695	14	67.7	3.5	3.0	0.2	695	14	67.7	3.5	3.0	0.2	12	0.0	0.0	0.0	0.0
Crackers	353	15	65.3	6.2	2.9	0.3	353	15	64.3	6.1	2.8	0.3	86	−1.0	0.2	0.0	0.0
Mixed Dishes—M/P/F	189	16	63.2	10.7	2.8	0.5	189	16	62.3	10.8	2.8	0.5	69	−0.9	0.3	0.0	0.0
Eggs	332	17	62.9	6.4	2.8	0.3	332	18	58.5	5.8	2.6	0.3	234	−4.4	0.9	−0.20	0.0
Condiments and Sauces	487	18	61.0	5.8	2.7	0.3	487	17	60.7	5.7	2.7	0.3	18	−0.3	0.1	0.0	0.0
Vegetables, excluding Potatoes	560	19	55.7	4.5	2.5	0.2	560	19	55.1	4.6	2.4	0.2	22	−0.6	0.4	0.0	0.0
White Potatoes	401	20	45.7	4.1	2.0	0.2	401	20	44.9	4.1	2.0	0.2	78	−0.8	0.2	0.0	0.0
Cooked Grains	299	21	44.5	4.9	2.0	0.2	299	21	44.5	4.9	2.0	0.2	0	0.0	0.0	0.0	0.0
Flavored Milk	253	22	38.1	4.8	1.7	0.2	253	22	38.1	4.8	1.7	0.2	0	0.0	0.0	0.0	0.0
Plant-Based Protein Foods	346	23	31.9	3.5	1.4	0.2	346	23	31.9	3.5	1.4	0.2	1	0.0	0.0	0.0	0.0
Mixed Dishes—Asian	89	24	30.0	7.0	1.3	0.3	89	24	30.0	7.0	1.3	0.3	1	0.0	0.0	0.0	0.0
Meats	220	25	29.9	3.7	1.3	0.2	220	25	29.9	3.7	1.3	0.2	5	0.0	0.0	0.0	0.0
Seafood	96	26	26.7	7.3	1.2	0.3	96	26	26.6	7.3	1.2	0.3	26	−0.1	0.0	0.0	0.0
Fats and Oils	460	27	24.0	1.6	1.1	0.1	460	27	24.0	1.6	1.1	0.1	31	0.0	0.0	0.0	0.0
Sweetened Beverages	787	28	22.9	2.4	1.0	0.1	787	28	22.6	2.3	1.0	0.1	23	−0.3	0.1	0.0	0.0
**Mean Sodium Intake (mg) of Children 6–11 Years of Age (*n* = 2193)**																	
**WWEIA Food Group**	**Actual Intake**						**Adjusted Intake**						**Delta Intake**				
**Sub Group Description**	**Cons**	**Rank**	**Mean**	**SE**	**Pct**	**SE**	**Cons**	**Rank**	**Mean**	**SE**	**Pct**	**SE**	**Cons**	**Mean**	**SE**	**Pct**	**SE**
Mixed Dishes—Pizza	548	1	286.2	29.5	9.4	1.0	548	2	206.7	21.3	6.8	0.7	547	−79.4	8.9	−2.6	0.3
Mixed Dishes—Mexican	362	2	214.7	25.0	7.1	0.8	362	7	168.1	19.9	5.5	0.6	347	−46.6	5.8	−1.5	0.2
Cured Meats/Poultry	687	3	197.0	13.0	6.5	0.4	687	3	197.0	13.0	6.5	0.4	1	0.0	0.0	0.0	0.0
Mixed Dishes—Sandwiches	436	4	193.6	17.9	6.4	0.6	436	5	181.	17.2	6.0	0.5	170	−12.4	1.9	−0.4	0.1
Breads, Rolls, Tortillas	1205	5	193.5	6.7	6.4	0.3	1205	4	192.74	6.6	6.4	0.3	85	−0.8	0.2	0.0	0.0
Mixed Dishes—Grain-based	606	6	185.5	13.0	6.1	0.4	606	8	163.0	10.6	5.4	0.3	324	−22.5	3.4	−0.7	0.1
Poultry	711	7	169.0	11.5	5.6	0.4	711	6	168.9	11.5	5.6	0.4	124	−0.1	0.0	0.0	0.0
Sweet Bakery Products	1034	8	129.8	6.8	4.3	0.2	1034	9	128.5	6.7	4.2	0.2	455	−1.4	0.2	0.0	0.0
Mixed Dishes—M/P/F	281	9	109.2	19.1	3.6	0.6	281	11	106.9	18.8	3.5	0.6	123	−2.3	0.6	−0.1	0.0
Cheese	723	10	108.3	9.7	3.6	0.3	1643	1	277.2	12.2	9.1	0.4	1349	168.9	11.7	5.6	0.4
Quick Breads and Bread Products	475	11	105.0	7.8	3.5	0.3	475	13	102.7	7.7	3.4	0.3	445	−2.3	0.2	−0.1	0.0
Savory Snacks	1048	12	103.5	5.3	3.4	0.2	1048	12	102.9	5.2	3.4	0.2	232	−0.6	0.1	0.0	0.0
Condiments and Sauces	867	13	98.4	9.9	3.2	0.3	867	14	97.5	9.9	3.2	0.3	32	−1.0	0.4	0.0	0.0
Mixed Dishes—Soups	253	14	95.7	12.5	3.2	0.4	253	15	95.2	12.3	3.1	0.4	6	−0.5	0.4	0.0	0.0
Milk	1274	15	89.2	3.2	2.9	0.1	1987	10	108.1	3.4	3.6	0.1	1646	18.92	0.9	0.6	0.0
Ready-to-Eat Cereals	861	16	82.6	5.2	2.7	0.2	861	16	82.6	5.2	2.7	0.2	24	−0.1	0.0	0.0	0.0
White Potatoes	610	17	67.5	5.4	2.2	0.2	610	17	65.7	5.2	2.2	0.2	105	−1.8	0.5	−0.1	0.0
Meats	388	18	59.2	6.3	2.0	0.2	388	18	59.2	6.3	2.0	0.2	5	0.0	0.0	0.0	0.0
Eggs	324	19	54.3	5.2	1.8	0.2	324	19	51.2	4.8	1.7	0.2	217	−3.1	0.7	−0.1	0.0
Vegetables, excluding Potatoes	792	20	50.4	4.9	1.7	0.2	792	21	49.7	4.8	1.6	0.2	40	−0.7	0.2	0.0	0.0
Flavored Milk	555	21	50.3	4.3	1.7	0.1	555	20	50.3	4.3	1.7	0.1	0	0.0	0.0	0.0	0.0
Cooked Grains	302	22	46.2	5.0	1.5	0.2	302	22	46.1	5.0	1.5	0.2	1	−0.1	0.1	0.0	0.0
Crackers	325	23	42.2	4.0	1.4	0.1	325	24	41.5	4.0	1.4	0.1	96	−0.7	0.1	0.0	0.0
Fats and Oils	740	24	42.1	3.5	1.4	0.1	740	23	42.1	3.5	1.4	0.1	62	−0.1	0.0	0.0	0.0
Sweetened Beverages	1467	25	42.0	2.0	1.4	0.1	1467	25	41.0	1.9	1.4	0.1	45	−1.0	0.5	0.0	0.0
Mixed Dishes—Asian	142	26	38.4	6.6	1.3	0.2	142	26	38.4	6.6	1.3	0.2	5	0.0	0.0	0.0	0.0
Plant-Based Protein Foods	475	27	35.7	3.3	1.2	0.1	475	27	35.7	3.3	1.2	0.1	1	0.0	0.0	0.0	0.0
Other Desserts	620	28	30.2	5.4	1.0	0.2	620	29	22.0	4.4	0.7	0.2	478	8.3	1.1	−0.3	0.0
**Mean Sodium Intake (mg) of Children 12–18 Years of Age (*n* = 2172)**																	
**WWEIA Food Group**	**Actual Intake**						**Adjusted Intake**						**Delta Intake**				
**Sub Group Description**	**Cons**	**Rank**	**Mean**	**SE**	**Pct**	**SE**	**Cons**	**Rank**	**Mean**	**SE**	**Pct**	**SE**	**Cons**	**Mean**	**SE**	**Pct**	**SE**
Mixed Dishes—Pizza	486	1	296.5	31.1	8.7	0.9	486	3	216.3	22.8	6.4	0.7	486	−80.2	8.7	−2.4	0.3
Mixed Dishes—Mexican	329	2	224.4	26.3	6.6	0.8	329	8	178.3	20.7	5.3	0.6	313	−46.1	6.3	−1.4	0.2
Cured Meats/Poultry	585	3	222.7	19.3	6.6	0.5	585	2	222.1	19.3	6.5	0.5	2	−0.6	0.5	0.0	0.0
Mixed Dishes—Sandwiches	374	4	210.6	20.2	6.2	0.6	374	6	191.9	18.2	5.7	0.5	174	−18.7	3.4	0.6	0.1
Breads, Rolls, Tortillas	1119	5	209.8	8.8	6.2	0.2	1119	4	209.0	8.7	6.2	0.2	85	−0.8	0.2	0.0	0.0
Mixed Dishes—Grain-based	464	6	200.5	14.8	5.9	0.4	464	7	179.7	14.1	5.3	0.4	244	−20.7	2.8	−0.6	0.1
Poultry	652	7	193.2	18.4	5.7	0.5	652	5	193.2	18.4	5.7	0.5	130	−0.1	0.0	0.0	0.0
Condiments and Sauces	889	8	146.7	13.5	4.3	0.4	889	9	145.5	13.4	4.3	0.4	41	−1.1	0.3	0.0	0.0
Mixed Dishes—M/P/F	288	9	133.4	19.7	3.9	0.6	288	10	128.8	19.6	3.8	0.6	100	−4.6	1.1	−0.2	0.0
Cheese	687	10	117.9	7.8	3.5	0.3	1565	1	300.1	12.1	8.8	0.4	1276	182.3	12.0	5.4	0.3
Savory Snacks	942	11	113.8	9.1	3.4	0.3	942	11	112.7	9.0	3.3	0.3	233	−1.1	0.2	0.0	0.0
Mixed Dishes—Soups	237	12	111.1	11.0	3.3	0.3	237	12	110.2	11.0	3.3	0.3	10	−0.9	0.5	0.0	0.0
Sweet Bakery Products	833	13	108.1	8.4	3.2	0.2	833	15	106.7	8.3	3.14	0.2	344	−1.4	0.2	0.0	0.0
Meats	424	14	107.7	9.8	3.2	0.3	424	14	107.7	9.8	3.2	0.3	14	0.0	0.0	0.0	0.0
White Potatoes	580	15	103.1	9.9	3.0	0.3	580	16	95.6	9.5	2.8	0.3	121	−7.5	1.6	−0.2	0.1
Mixed Dishes—Asian	174	16	94.4	20.1	2.8	0.6	174	17	94.4	20.1	2.8	0.6	11	0.0	0.0	0.0	0.0
Milk	973	17	90.0	4.7	2.7	0.1	1779	13	109.2	4.7	3.2	0.2	1453	19.2	1.0	0.6	0.0
Quick Breads and Bread Products	297	18	81.8	7.4	2.4	0.2	297	18	80.2	7.3	2.4	0.2	282	−1.6	0.2	−0.1	0.0
Ready-to-Eat Cereals	582	19	75.2	5.2	2.2	0.2	582	19	75.2	5.2	2.2	0.2	18	0.0	0.0	0.0	0.0
Eggs	333	20	66.2	5.7	2.0	0.2	333	21	62.6	5.3	1.9	0.2	211	−3.6	0.6	−0.1	0.0
Fats and Oils	700	21	63.2	7.4	1.9	0.2	700	20	63.0	7.4	1.9	0.2	86	−0.2	0.0	0.0	0.0
Sweetened Beverages	1404	22	59.4	4.7	1.8	0.1	1404	22	58.5	4.8	1.7	0.1	40	−0.9	0.2	0.0	0.0
Vegetables, excluding Potatoes	803	23	57.6	7.1	1.7	0.2	803	23	56.9	7.1	1.7	0.2	28	−0.7	0.2	0.0	0.0
Cooked Grains	325	24	51.8	5.2	1.5	0.2	325	24	51.8	5.2	1.5	0.2	0	0.0	0.0	0.0	0.0
Crackers	229	25	43.1	6.4	1.3	0.2	229	25	42.6	6.3	1.3	0.2	69	−0.5	0.1	0.0	0.0
Plant-Based Protein Foods	344	26	35.7	5.0	1.1	0.1	344	26	35.7	5.0	1.1	0.1	1	0.0	0.0	0.0	0.0

^1^ To a 1% contribution of daily intake of sodium; ^2^ Nutrients from milk, cheese, and yogurt for non-dairy foods are added to the nutrients in the milk, cheese, and yogurt food categories, respectively. For non-dairy foods the nutrients displayed are only for the milk, cheese, and yogurt in the non-dairy food. Abbreviations: Cons = consumers, M/P/F = meat/poultry/fish; SE = standard error; Pct = percent contribution to energy intake or specific nutrient intake, as appropriate.
